# Health inequity in workers of Latin America and the Caribbean

**DOI:** 10.1186/s12939-020-01228-x

**Published:** 2020-07-01

**Authors:** Michael Silva-Peñaherrera, María Lopez-Ruiz, Pamela Merino-Salazar, Antonio Ramón Gómez-García, Fernando G. Benavides

**Affiliations:** 1 CIBER Epidemiología y Salud Pública (CIBERESP), Center for Research in Occupational Health (CISAL), Universitat Pompeu Fabra, IMIM (Hospital del Mar Medical Research Institute), Edificio PRBB, Doctor Aiguader, 88, 08003 Barcelona, Spain; 2 International University SEK, Quito, Ecuador; 3 Universidad Espiritu Santo, Guayaquil, Ecuador

**Keywords:** Health inequalities, Occupational health; self-perceived health, Inequity

## Abstract

**Background:**

Latin America and the Caribbean (LAC) is the world’s most inequitable region in terms of wealth distribution. The full scale of social inequalities in health has been hidden by the lack of reliable data. This study aimed to measure and compare health inequalities in the working population within and between 15 countries of LAC.

**Methods:**

A sample of 180,163 workers aged 18 years and older was drawn from the most recent national surveys of working conditions or health in 15 LAC countries. Poor self-perceived health (P-SPH) was used as a health indicator, and age, education level, and occupational category as inequality stratifiers. We calculated four measures: absolute and relative population-attributable risks, the Kuznets and weighted Keppel indexes.

**Results:**

P-SPH prevalence ranged from 9% in men from Uruguay to 50% in women from Nicaragua. It was higher in women than in men in most countries. A clear gradient was shown, in which young people in non-manual skilled jobs and high education had the lowest prevalence. Nearly 45% of cases that reported P-SPH among men and 35% among women could be avoided if all the groups received a higher level of education. Also, approximately 42% of P-SPH reported by men and 31% by women could be avoided if they all shared the working and employment conditions of non-manual skilled jobs.

**Conclusions:**

Wide health inequalities were found between occupational and educational groups in LAC. However, country borders appear to be an even more important stratifier in the production of health inequalities. Urgent interventions to improve worker’s health are needed in countries where prevalence of poor self-perceived health is high. Strengthening occupational health surveillance system in LAC countries should become a priority, in order to track the interventions to reduce occupational health inequity.

## Introduction

The economic and social growth of Latin America and the Caribbean (LAC) has been hampered by enormous social inequalities in the last decades. As reported, LAC is the most inequitable region in the world in terms of wealth distribution [1, 2]. The size of these gaps is much bigger than is perceived by the population in general [3], and examination of the full impact of this inequity in the population’s health in the region has been limited by the lack of reliable data [4].

According to a growing body of scientific evidence, there is strong evidence of a significant association between poor social, economic, and political conditions and social inequalities in self-perceived health [5, 6]. Based on conceptual model of the WHO Commission on Social Determinants of Health, the conditions in which people are born, grow, live, work and age are linked to position on the social hierarchy [7]. These condition are generally responsible for health inequities, yet the magnitude and the slope of the gradient can vary within and between countries. Studying the role of paid work, as one of the most important social determinants of health [8], especially in low and middle-income countries, will help us better understand those inequalities. However, most of these evidence were focused on the general population, and the few studies that have attempted to examine the contribution of working and employment conditions in health inequalities were mostly carried out in wealthy countries [9]. Inequality worldwide has not been reduced but has risen over the last three decades and continuies to grow, across and especially within countries [10].

Paid work has an important effect on population health and health inequalities, for both the working and general population [11]. The working population is the main producer of goods and services and contributes to the economic growth of the countries. Furthermore, paid work is the principal income source for the overwhelming majority of people and is the main means of both wealth redistribution [12] and access to social protections. In LAC, 280 million people (40% of them women) work, and 53.1% of them have informal employment [13].

Occupational risk factors related to workplace environment, job tasks, psychosocial demands, and other conditions directly affect workers’ health and contribute significantly to health inequality [14, 15], especially in low- and middle-income countries [7], where failure to enforce regulations has resulted in poor working and employment conditions. Annually, worldwide, 2.02 million people die due to work-related diseases and 318,000 people due to occupational injuries [16]. Globalization’s reliance on “labor flexibility” and outsourcing has transferred worker health costs from high-income to low- and middle-income countries [17, 18], increasing the health gap across countries and world regions. In LAC, fatal and non-fatal occupational injury rates are five times higher than the world’s average [19].

Self-perceived health (SPH) has increasingly been used to measure health status since this measure has been found to be reliable, valid, simple and cost-effective [20]. This measure summarizes much information in one question. Consequently, most national surveys that include an assessment of health routinely include it in theirs questionnaires, rating health from poor to excellent by a four or five-point Likert scale.

The objective of this study was to measure and compare health inequalities in working populations within and between 15 countries in LAC.

## Methods

### Population and data source

This cross-sectional analysis is based on the most recent national surveys of health or working conditions from a representative sample of 15 countries in LAC: Argentina, Brazil, Chile, Colombia, Costa Rica, Ecuador, El Salvador, Guatemala, Honduras, Nicaragua, Mexico, Panama, Peru, Puerto Rico, and Uruguay. We selected these 15 LAC countries because they have a national survey on a representative sample conducted between 2012 and 2018, and which included the SPH indicator. For Mexico instead of the national health survey, which does not include the SPH question, we used the world value survey [21]. The questionnaires used in each national survey were administered in face-to-face interviews at the workers’ homes (except in Puerto Rico, where the interviews were conducted by telephone).

The micro-data, methodology, and details of each survey were downloaded from the official website when they were available online or requested from the organization in charge in the country when they were not. Sources and the population’s general characteristics from each survey are described in Supplementary Table A.

For the purposes of this study, we selected only working individuals (who affirmed having worked at least 1 h in the week prior to the interview) aged 18 years and older, since most surveys use this age as a minimum for inclusion. The final sample under analysis included 180,163 workers engaged in the formal or informal economy, and from all economic sectors with the exception of armed forces occupations. The representation by country was as follows: Argentina, 20,060; Brazil, 89,750; Chile, 3648; Colombia, 16,812; Costa Rica, 153; Ecuador, 33,554; El Salvador, 1507; Guatemala, 1510; Honduras, 1507; Mexico, 996; Nicaragua, 1501; Panama, 1505; Peru, 3105; Puerto Rico, 1515; and Uruguay, 1691. Survey data were weighted using the specific expansion factor or the weight factor from each survey, except for Mexico and Puerto Rico where no factor was provided. Detailed descrption of the survey methodology is available elsewhere [21–28].

### Health indicators

Self-perceived health (SPH) was selected as a health indicator. It was collected using a four-point Likert scale in Colombia and Mexico, and a five-point Likert scale in the rest of the countries studied. The response categories for each survey were dichotomized, so that a response of “fair” or less indicated poor SPH (P-SPH) (Supplementary Table B).

### Equity stratifiers

Data were disaggregated separately for women and men in the three equity stratifiers: age (grouped as 15–24 years, 25–44 years, 45–65 years, and over 65 years); education level (less than low being “less than elementary school”, low being “elementary school”, middle being “high school”, and high being “more than high school”); and occupational categories, where the nine major categories of the International Standard Classification of Occupations (ISCO) [29] were collapsed into four categories: skilled non-manual (managers, professionals, technicians, and associate professionals), non-manual non-skilled (clerical support workers, service and sales workers), skilled manual (skilled agricultural, forestry and fishery workers, craft and related trades workers, plant and machine operators, and assemblers), and non-skilled manual (elementary occupations).

### Inequality measures and data analysis

First, we calculated the prevalence and 95% confidence intervals (CIs) of P-SPH for each of the three equity stratifiers for every country (Supplementary Table C). Then, following the recommendations of the WHO Handbook on health inequality monitoring [30], we calculated four measures of inequality [31]. For all, the reference category used was the healthiest group: 15–24 for age, high educational level for education, and non-manual skilled jobs for the occupational category.

Of the four measures, the first two estimate the magnitude of difference and the proportional difference between the healthiest group and the country’s mean. These are the population-attributable risk (PAR), and the population-attributable risk percentage (PAR%), with respect to the country’s mean prevalence. These measures represent the percentage of the population that would not declare P-SPH if the entire working population shared the condition of the most privileged group (see Table 1 footnote).

**Table 1:** Population-attributable risk of inequality by age, education level, and occupational categories

	Women						Men					
	Age groups		Education Level		Occupational categories		Age groups		Education Level		Occupational categories	
Country	Population attributable risk	Population attributable risk(%)	Population attributable risk	Population attributable risk(%)	Population attributable risk	Population attributable risk(%)	Population attributable risk	Population attributable risk(%)	Population attributable risk	Population attributable risk(%)	Population attributable risk	Population attributable risk(%)
Argentina	10.4	58.2	8.6	47.8	–	–	8.5	60.5	7.6	53.6	–	–
Brazil	10.7	42.4	12.4	49.1	10.8	42.7	10.7	48.0	11.8	53.1	9.6	43.1
Chile	21.9	81.5	7.3	27.6	6.6	25.0	7.5	52.0	8.4	58.2	6.3	43.6
Colombia	7.8	43.0	7.9	43.6	7.6	41.6	7.9	58.5	7.7	57.0	7.2	53.3
Costa Rica	16.7	60.0	7.3	26.3	14.1	50.9	9.3	36.3	15.9	62.1	12.0	46.8
Ecuador	12.5	31.9	13.0	33.3	13.4	34.1	10.8	32.2	13.4	39.7	13.1	39.0
El Salvador	20.1	63.8	7.4	23.6	4.6	14.6	15.3	57.0	16.8	62.6	15.7	58.5
Guatemala	4.1	20.9	7.6	38.9	6.2	31.8	5.7	23.6	17.0	70.2	18.3	75.3
Honduras	7.8	18.1	22.8	52.9	6.0	13.8	25.0	57.1	17.0	38.7	20.2	46.0
Mexico	13.3	55.3	14.1	58.9	17.3	72.2	4.5	19.4	12.0	52.3	4.8	20.8
Nicaragua	12.5	26.0	15.0	31.2	10.0	20.9	15.8	36.5	11.7	26.9	13.4	30.8
Panama	16.3	54.3	4.5	14.8	5.4	17.9	11.2	48.7	6.6	28.9	6.3	27.3
Peru	14.4	33.0	11.6	26.5	2.2	5.1	14.0	40.4	8.6	24.8	9.8	28.5
Puerto Rico	18.1	73.7	2.7	10.9	–	–	10.4	57.5	5.5	30.6	–	–
Uruguay	10.4	68.2	5.9	38.8	5.5	36.4	1.8	19.8	2.3	24.7	3.4	36.5

Population-attributable risk = Conutry prevalence of SPH minus healthiest-group prevalence of SPH

Population-attributable risk(%) = Population attributable risk divided by the population mean × 100

The third measure, the relative Kuznets index, is the ratio between each group’s P-SPH prevalence and that of the reference groups. For exmaple this would be the P-SPH prevalence of the low educational level group divided by the P-SPH prevalence of the reference group, and was calculated for each equity stratifier.

The fourth measure was the weighted Keppel index. This measure is the absolute difference between the P-SPH prevalence of each group and the country’s P-SPH average prevalence. The absolute value of these differences is multiplied by the population weight of each group, and the sum of these weighted differences is divided by the country’s P-SPH prevalence and multiplied by 100 [32]. The magnitude reflects the average relative gap between the P-SPH prevalence of each group in the category and the country’s mean (Fig. 2). The 95% CIs were calculated for both the P-SPH prevalence and for the Kuznets index (See footnotes in supplementary Table D).

## Results

### Prevalence of poor self-perceived health

**Fig. 1:**
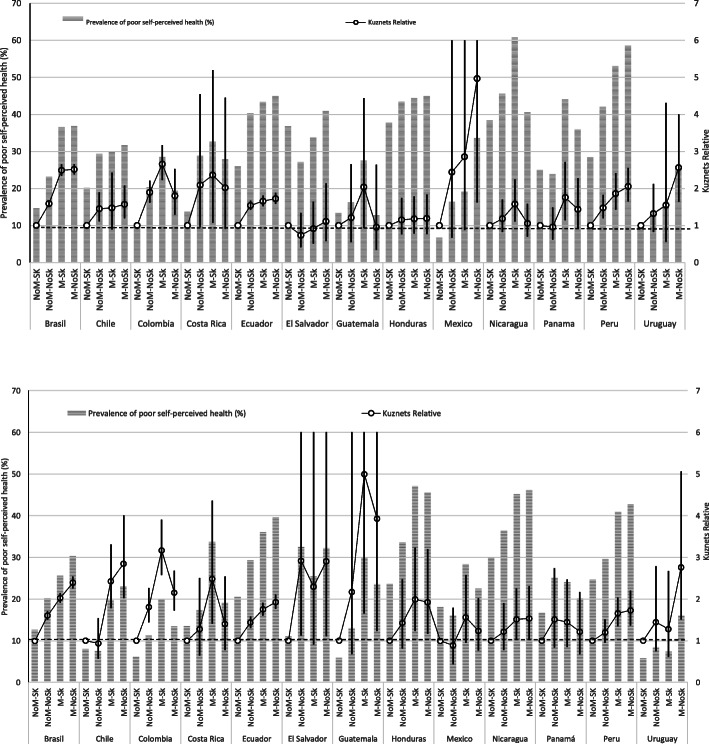
Prevalence of poor self-perceived health (%) and Kuznets Relative index 95% Cis in occupational categories stratified by sex (women). Prevalence of poor self-perceived health (%) and Kuznets Relative index 95% CIs in occupational categories stratified by sex (men)

Among the different countries, P-SPH prevalence ranged from 9.2% in men from Uruguay to 48% in women from Nicaragua. It was consistently higher in women than in men, except in Guatemala and Honduras, where it was similar for the two sexes. A clear gradient was observed with both age and educational level, as P-SPH increased with age and decreased with years of study (Fig. 1). Regarding occupational categories, non-manual skilled workers had the lowest prevalence of P-SPH in all countries, while manual skilled and manual non-skilled had the highest.

### Measures of inequity

Overall, the widest gap was found among the age groups, followed by the educational levels and occupational categories. Regarding the PAR (Table 1), women from Chile (21.9%) and men from Honduras (25.0%) showed the highest magnitude by age. The PAR% had a wide range among countries: the lowest magnitude by age was found in men from Uruguay (19.8%) and the highest in women from Chile (81.5%). Stratified by educational level, the PAR% ranged from 10.9% in women from Puerto Rico to 70.2% in men from Guatemala; by occupational category, it ranged from 5.1% in women from Peru to 75.3% in men from Guatemala. The relative difference according to educational level and occupational category was higher in men, with some clear exceptions, as Mexico and Costa Rica showed the opposite tendency.

The P-SPH prevalence in the highly educated group (the reference group) ranged from 5.8 in men from Colombia to 33.0 in women form Nicaragua (Fig. 1). In the occupational categories, the P-SPH prevalence of the non-manual skilled group (reference group) ranged from 5.8% in men from Uruguay to 37.9% in women from Nicaragua (Fig. 1).

In regard to the Kuznets index, the highest value was associated with the variable age in men from Chile (7.9), followed by education level in women from Mexico (8.1). In some countries, the category groups on the extremes had wide confidence intervals, due to the small sizes of these groups. Overall, the Kuznets values were slightly higher in women than in men. A clear gradient was observed in the three dimensions. By occupation, manual non-skilled workers presented the highest Kuznets index in most countries, reaching 5.0 in women from Mexico (Fig. 1).

**Fig. 2:**
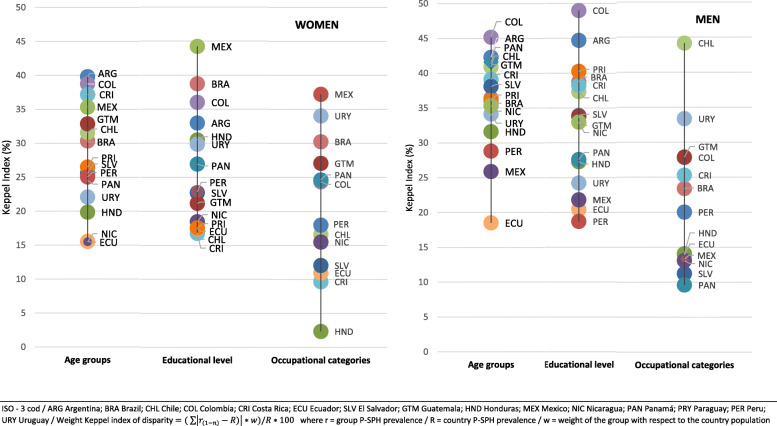
Health inequality in the working populations according to age, education level, and occupational category, stratified by sex (weighted Keppel index)

The weighted Keppel index was higher for men than for women. The largest gap among countries was observed between occupational categories, where the index ranged from 2.4 for women from Honduras to 44.2 for men from Chile. By age, Ecuador had the lowest values (15.6 for women and 18.5 for men), while Colombia had the highest (38.3 for women and 45.2 for men). By level of studies, Ecuador were the country with the lowest index values (17.4 for women and 20.5 for men), and Argentina and Colombia had the highest for men (44.7 and 49.0, respectively) (Fig. 2).

## Discussion

This study revealed that, on average, around 45% of men and 35% of women workers that reported P-SPH from the 15 countries could avoid it if all the groups received a high level of education, and that around 42% of men and 31% of women could avoid P-SPH if they had the working and employment conditions of the workers with non-manual skilled jobs. This magnitude of inequity among groups indicates the scale of the possible improvement in the countries, and demonstrates what is feasible for other social groups to attain.

The most privileged group had the best health and it would be desirable for the other groups to be equally healthy. Nevertheless, the results show that the widest range of P-SPH observed is among the reference (healthiest) groups in the different countries. Hence, it would also be desirable to lessen the gap between countries. For example, in education level, the most privileged group in Nicaragua (those whose level of education was high) showed similar or higher prevalence of P-SPH than the worst groups in Argentina, Colombia, Chile, Guatemala, and Uruguay (whose level of education was less than low). Likewise, by occupation, the most privileged group of women in Nicaragua and Honduras had a higher prevalence of P-SPH than the worst category in most of the countries in this study. This means that most of the workers in the least-favored occupational category in Uruguay or Chile are in better health than most of the workers in the most-favored occupational category in Honduras or Nicaragua.

Actually, the fact that some countries have low inequity indexes should not obscure the results since, overall, the higher the prevalence of P-SPH, the lower the inequity index. For example, Nicaragua’s and Honduras’ Keppel index values related to occupation are under 15%, and these two countries show the highest prevalence of P-SPH in the region, while Uruguay’s Keppel index is over 33% in the same category, even though it has the lowest P-SPH prevalence. In general, this means that inequity across social groups is low, but this is mainly because most of the working population reports P-SPH. Therefore, health conditions in those countries where more than one-third of the total working population reported P-SPH demand urgent intervention, with special focus on the most vulnerable groups, which in some countries reach a P-SPH prevalence of over 60%.

The worker’s health gaps among countries were bigger than those associated with differences in sex, age, education level, or occupation, suggesting that workers’ health is determined to a greater extent by the country where they work. Similar patterns of inequity are observed with diverse health indicators among the general population worldwide [33], supporting the idea that borders generate more inequality than any other variable and could be related to social, economic, and political factors but, most essentially, to international differences in regulations and laws [34]. An alternative explanation could be ascribed to the methodologies used in the various countries’ surveys. It is known that there are differences among working-condition surveys [4], while health surveys are more standardized [35]. However, in Central America, where the methodology was the same, we found a wide difference among countries’ prevalence of P-SPH, ranging from 19.6 in women from Guatemala to 48.0 in women from Nicaragua.

Regarding gender inequity, as many studies have shown [36], the prevalence of P-SPH in the overwhelming majority of the countries was higher in women (29% of women and 24% of men in LAC as a whole). This has also been found in Europe, where women over 16 years old reported 3.7% more P-SPH than men (30% of women and 26.3% of men). Regardless, the inequity gap was larger among men in the three equity stratifiers. The highest relative differences by sex were found in Chile, in which the prevalence in women was 85% higher than in men and the lowest differences were in Mexico, where P-SPH was 4.4% higher in women. Guatemala and Honduras were an exception; nevertheless, the prevalence of P-SPH was almost the same for both sexes. These results are probably influenced by cultural factors that these neighboring countries (Mexico, Guatemala, and Honduras) share and that are mainly linked to the beliefs and behavior related to gender roles [37]. The patriarchal culture still predominates and exposes men to risk behaviors that could be detrimental to their health [38]. Another possible explanation could be the high index of criminal violence and drug trafficking in these countries, which mainly affects men. The SPH could be influenced by differences in culture, ethnicity, and sex [39]. To better understand these results, more studies of these possible influences are needed.

This study, as any other, has limitations, mainly related to the comparability of the data. The most recent national surveys on working conditions and health were used. We consider them to have the best and most reliable data on SPH available in each country. However, as the data come from different countries and therefore from different sources, the education categories may vary slightly between countries, as can the scale used to collect the SPH data. Additionally, the surveys were carried out in different years, between 2012 and 2018, and this could affect results. However, this range is relatively narrow, and the overall socioeconomic situation in LAC was stable in these years [40]. Anyway, the comparison between countries must be made with caution. Though not all countries were included in this study because no data were found for Bolivia, Paraguay, Venezuela and others, the final sample does represent most of the working population in the region. Finally, ethnic group was not used as an equity stratifier because the data were not included in all the countries‘surveys. Nevertheless, to our knowledge, this is the first study to use large national datasets from LAC countries to study health inequity and provide the first cross-country comparisons of the health status in the working population. SPH has been shown to be a strong and independent predictor of mortality and strongly associated with morbidity [41], even demonstrating better reliability than objective measures of morbidity and psychological well-being in some studies [42].

Furthermore, the final datasets and results will ultimately be made available to the scientific community for future, high-quality studies, useful to researchers and policy makers alike. The micro-data used for this analysis are a small portion of the information available from the countries’ surveys. These data are not always accessible and, in many cases, the shift to open access takes several years. So, given the relevance of this information to the region, it is essential to allow access to this data to researchers from different fields and countries, as well to find mechanisms to ensure the comparability of questions and methodology among the countries’ surveys to allow comparison and monitoring of changes over time.

These results show vulnerable groups within countries, but moreover, they show vulnerable countries in the region. Many countries are now focusing on reducing this gap [43]. Additionally, five Sustainable Development Goals of the United Nations 2030 Agenda are related to the reduction of inequalities. SDG 10 “to reduce inequality within and among countries”, SDG 1 “to end poverty”, SDG 4 “to ensure inclusive and equitable quality education”, SDG 5 “to achieve gender equality” and SDG 3 “ensure healthy lives and promote well-being for all at all ages” [44]. Even though many more efforts will be necessary to reduce all systematic differences in health, mainly leveling up all members of society to the health of the most advantaged group and in the region [45].

## Conclusion

This analysis represents a first step in monitoring occupational health inequity in the region. The dissemination of these findings could raise the awareness of members of society, local governments and international organizations motivating them to take urgent actions to address health inequalities in the most unequal region of the planet. Public policy aimed at reducing health inequity in LAC countries must ensure decent working and employment conditions for all. According to our results, these policies should target unskilled and less educated occupational groups, particularly women.

Periodic updates to track changes in working conditions over time and the progress of health improvements in closing the gaps among social groups will be essential [46]. In this regard, taking into account the specific situation in each country, goverments should promote periodic working, employment and health surveys as the main sources for monitoring health system. Strong national health monitoring systems are fundamental. International and regional collaboration will be necessary to manage this challenge.

## Supplementary information

**Additional file 1: Supplementary Table A.** Distribution (percentage) of the population sample by country and sex (%) according to age group, education level, and occupation. **Supplementary Tabla B.** Self-perceived health scale in surveys from 15 countries. **Supplementary Table C.** Prevalence of poor self-perceived health (%) and 95% confidence interval. **Supplementary Table D.** Prevalence of poor self-perceived health (%) and Kuznets relative index with 95% confidence interval. **Continue… Supplementary Table D.** Prevalence of poor self-perceived health (%) and Kuznets relative index with 95% confidence interval

## Data Availability

The datasets that support the findings of this study are available from the organization in charge in each of the 15 countries or available online in the country official website. Data are however available from the authors upon reasonable request.

## References

[1] Ravallion M (2014). Income inequality in the developing world. Science..

[2] World Bank. World development indicators 2017. Washington, D.C.; 2017. 197 p. Available from: www.worldbank.org.

[3] Norton MI, Ariely D (2011). Building a Better America—One Wealth Quintile at a Time. Perspect Psychol Sci.

[4] Merino-Salazar P, Artazcoz L, Campos-Serna J, Gimeno D, Benavides FG. National working conditions surveys in Latin America: comparison of methodological characteristics. Int J Occup Environ Health. 2015;21(3):266–274. Available from: http://www.ncbi.nlm.nih.gov/pubmed/26079314 (Accesed 17 Aug 2016). 10.1179/2049396715Y.0000000004 PMC4597016 26079314

[5] Bouchard L, Albertini M, Batista R, de Montigny J (2015). Research on health inequalities: a bibliometric analysis (1966–2014). Soc Sci Med.

[6] Cash-Gibson L, Rojas-Gualdrón DF, Pericàs JM, Benach J. Inequalities in global health inequalities research: A 50-year bibliometric analysis (1966-2015). PLoS One. 2018;13(1). [cited 2020 Apr 23] Available from: 10.1371/journal.pone.0191901. 10.1371/journal.pone.0191901 PMC5792017 29385197

[7] Benach J, Muntaner C, Solar O, Santana V, Quinlan M (2010). Introduction to the who commission on social determinants of health employment conditions network (Emconet) study, with a glossary on employment relations. Int J Heal Serv.

[8] Marmot M (2017). The health gap: doctors and the social determinants of health. Scand J Public Health.

[9] Campos-Serna J, Ronda-Pérez E, Artazcoz L, Moen BE, Benavides FG (2013). Gender inequalities in occupational health related to the unequal distribution of working and employment conditions: a systematic review. Int J Equity Health.

[10] Alvaredo F, Chancel L, Piketty T, Saez E, Zucman G. World inequality report 2018. Vol. 70, Tijdschrift voor Geneeskunde. 2017. Available from: https://wir2018.wid.world/files/download/wir2018-full-report-english.pdf.

[11] Bambra C (2011). Work, worklessness and the political economy of health inequalities. J Epidemiol Community Heal.

[12] Bambra C. Work, Worklessness, and the Political Economy of Health [Internet]. Work, Worklessness, and the Political Economy of Health. Oxford University Press; 2011 [cited 2018 May 8]. 1–264 p. Available from: http://www.oxfordscholarship.com/view/10.1093/acprof:oso/9780199588299.001.0001/acprof-9780199588299. 10.1136/jech.2009.102103 21282147

[13] International Labour Organization. Women and men in the informal economy: a statistical picture (third edition). Geneva:ILO; 2018.

[14] Benavides FG, Benach J, Roman C, Diez-Roux AV, Roman C (2000). How do types of employment relate to health indicators? Findings from the second European survey on working conditions. J Epidemiol Community Health.

[15] Benach J, Vives A, Amable M, Vanroelen C, Tarafa G, Muntaner C. Precarious Employment: Understanding an Emerging Social Determinant of Health. Annu Rev Public Health. 2014;35(1):229–253. [cited 2018 Sep 7] Available from: http://www.annualreviews.org/doi/10.1146/annurev-publhealth-032013-182500. 10.1146/annurev-publhealth-032013-182500 24641559

[16] International Labour Office. ILO introductory report: global trends and challenges on occupational safety and health: XIX World Congress on Safety and Health at Work [Internet]. Istanbul, Turkey: International Labour Organization; 2011. 11–15 p. Available from: https://www.ilo.org/wcmsp5/groups/public/@ed_protect/@protrav/@safework/documents/publication/wcms_162662.pdf.

[17] KAWACHI I. Globalization and Workers’ Health. Ind Health. 2008;46(5):421–3. [cited 2018 Sep 7] Available from: http://joi.jlc.jst.go.jp/JST.JSTAGE/indhealth/46.421?from=CrossRef. 10.2486/indhealth.46.421 18840930

[18] Lucchini RG, London L (2014). Global occupational health: current challenges and the need for urgent action. Ann Glob Heal [Internet].

[19] Takala J, Hämäläinen P, Saarela KL, Yun LY, Manickam K, Jin TW (2014). Global estimates of the burden of injury and illness at work in 2012. J Occup Environ Hyg.

[20] Benyamini Y, Blumstein T, Murad H, Lerner-Geva L (2011). Changes over time from baseline poor self-rated health: For whom does poor self-rated health not predict mortality?. Psychol Health..

[21] Inglehart R, Haerpfer C, Moreno A, Welzel C, Kizilova K, Diez-Medrano J, et al. World values survey: round six - country-pooled Datafile version [internet]. World Values Survey: Round Six - Country-Pooled Datafile Version. Madrid; 2014 [cited 2020 Feb 20]. Available from: http://www.worldvaluessurvey.org/WVSDocumentationWV6.jsp.

[22] INDEC (2013). Encuesta Nacional de Factores de Riesgo 2013 Documento para la utilización de la base de datos usuario [Internet]. Buenos Aires.

[23] IBGE. Pesquisa Nacional de Saúde - PNS [Internet]. 2013 [cited 2020 Feb 20]. Available from: https://www.ibge.gov.br/estatisticas/sociais/saude/9160-pesquisa-nacional-de-saude.html?edicao=9161&t=conceitos-e-metodos.

[24] Departamento de Epidemiologia - Ministerio de Salud de Chile. ENCAVI – Encuesta Nacional de Calidad de Vida y Salud [Internet]. 2016 [cited 2020 Feb 20]. Available from: http://epi.minsal.cl/encuesta-encavi/.

[25] DIMPE -DANE. Encuesta Nacional de Calidad de Vida - ENCV COLOMBIA. 2017.

[26] INEC. Encueta Nacional de Salud y Nutricion 2012. 2012 [cited 2020 Feb 20]. Available from: https://www.ecuadorencifras.gob.ec/salud-salud-reproductiva-y-nutricion.

[27] Center for Disease Control and Prevention (CDC). BRFSS Survey Data and Documentation. 2017 [cited 2020 Feb 20]. Available from: https://www.cdc.gov/brfss/annual_data/annual_2017.html.

[28] Martinez Iñigo D. Encuesta sobre condiciones de trabajo seguridad y salud laboral en Uruguay. 2013. 133 p. Available from: http://www.oiss.org/estrategia/IMG/pdf/Encuesta_Uruguay.pdf.

[29] International Labour Organization. International Standard Classification of Occupations ISCO-08. Vol. I. Geneva; 2012. 433 p.

[30] World Health Organization., World Health Organization. Handbook on health inequality monitoring: with a special focus on low- and middle-income countries. WHO Library Cataloguing-in-Publication Data Handbook. Geneva; 2013 Aug. Available from: www.who.int.

[31] Hosseinpoor AR, Bergen N, Schlotheuber A, Grove J (2018). Measuring health inequalities in the context of sustainable development goals. Bull World Health Organ.

[32] Keppel K, Pamuk E, Lynch J, Carter-Pokras O, Kim I, Mays V (2005). Methodological issues in measuring health disparities. Vital Heal Stat.

[33] Max Roser. Global Inequality of Opportunity - Our World in Data. 2019 [cited 2019 Aug 30]. Available from: https://ourworldindata.org/global-inequality-of-opportunity.

[34] International Labour Organization. Working Conditions Laws Database. 2012 [cited 2019 Sep 30]. Available from: http://www.ilo.org/dyn/travail.

[35] International household Survey Network. Standardized health survey modules. 2014 [cited 2019 Sep 30]. Available from: http://www.ihsn.org/health-modules.

[36] Merino-Salazar P, Artazcoz L, Cornelio C, Iñiguez MJI, Rojas M, Martínez-Iñigo D (2017). Work and health in Latin America: results from the working conditions surveys of Colombia, Argentina, Chile, Central America and Uruguay. Occup Environ Med.

[37] Aguilar Montes de Oca, Yessica Paola; Valdez Medina, José Luis ; González-Arratia López-Fuentes, Norma Ivonne; González Escobar S Los roles de género de los Hombres y las mujeres en el México contemporaneo In: Enseñanza e Investigación en Psicología 2013. p. 207–224.

[38] Gutmann MC (2006). The meanings of macho being a man in Mexico City.

[39] Ai AL, Carretta HJ, Aisenberg E (2017). Cultural Strengths of Latino-American Subgroups: Differential Associations With Their Self-Rated Mental and Physical Health. J Cross Cult Psychol.

[40] Comisión Económica para América Latina y el Caribe (CEPAL). La ineficiencia de la desigualdad. 2018. Available from: https://repositorio.cepal.org/bitstream/handle/11362/43566/1/S1800302_es.pdf. Accessed 20 Nov 2019.

[41] Mossey JM, Shapiro E. Self-Rated Health: A Predictor of Mortality Among the Elderly. 1982 [cited 2019 Jan 21]; Available from: https://www.ncbi.nlm.nih.gov/pmc/articles/PMC1650365/pdf/amjph00655-0034.pdf. 10.2105/ajph.72.8.800 PMC1650365 7091475

[42] Burström B, Fredlund P (2001). Self rated health: Is it as good a predictor of subsequent mortality among adults in lower as well as in higher social classes?. J Epidemiol Community Health..

[43] World Bank LAC (2013). Shifting gears to accelerate shared prosperity in Latin America and the Caribbean.

[44] The United Nations Development Programe (2012). Sustainable Development Goals.

[45] Whitehead M, Dahlgren G. Concepts and principles for tackling social inequities in health : Levelling up Part 1. World Heal Organ Eur. 2007;1:20. Available from: http://www.euro.who.int/__data/assets/pdf_file/0010/74737/E89383.pdf.

[46] World Health Organization. Closing the health equity gap: policy options and opportunities for action. WHO Library Cataloguing-in-Publication Data Closing. Geneva; 2013. Available from: http://www.equityhealthj.com/content/pdf/1475-9276-11-59.pdf.

