# Supplementary material for: Health inequity in workers of Latin America and the Caribbean
Source: Int J Equity Health. 2020 Jul 1;19:109. doi: 10.1186/s12939-020-01228-x (PMC7329389; doi:10.1186/s12939-020-01228-x)
Supplement: Supplementary file 1 — Additional file 1: Supplementary Table A. Distribution (percentage) of the population sample by country and sex (%) according to age group, education level, and occupation. Supplementary Tabla B. Self-perceived health scale in surveys from 15 countries. Supplementary Table C. Prevalence of poor self-perceived health (%) and 95% confidence interval. Supplementary Table D. Prevalence of poor self-perceived health (%) and Kuznets relative index with 95% confidence interval. Continue… Supplementary Table D. Prevalence of poor self-perceived health (%) and Kuznets relative index with 95% confidence interval [file 12939_2020_1228_MOESM1_ESM.docx]

**SUPPLEMENTARY MATERIAL**

**Supplementary Table A. Distribution (percentage) of the population sample by country and sex (%) according to age group, education level, and occupation**

|  |  |  |  | Age groups (%) | | | |  | Educational Level (%) | | | |  | Occupational categories (%) | | | | |
| --- | --- | --- | --- | --- | --- | --- | --- | --- | --- | --- | --- | --- | --- | --- | --- | --- | --- | --- |
|  |  |  |  |  |  |  |  |  |  |  |  |  |  | Non-manual | | Manual | | |
| **Country / Survey (year)** | **Sex** | **n** |  | <25 | 25­–44 | 45–64 | >65 |  | >Low | Low | Middle | High |  | Skilled | Non-skilled | Skilled | Non-skilled | |
| **Argentina** |  |  |  |  |  |  |  |  |  |  |  |  |  |  |  |  |  | |
| Encuesta nacional de Factores de Riesgo (2013) | Women | 5.940 |  | 16.6 | 48.0 | 29.6 | 5.8 |  | 7.0 | 33.7 | 24.9 | 34.5 |  | - | - | - | - | |
|  | Men | 14.120 |  | 12.6 | 50.9 | 31.7 | 4.8 |  | 6.4 | 34.9 | 27.9 | 30.8 |  | - | - | - | - | |
| **Brazil** |  |  |  |  |  |  |  |  |  |  |  |  |  |  |  |  |  | |
| Pesquisa Nacional de Saúde  (2013) | Women | 38,938 |  | 15.4 | 52.6 | 30.0 | 1.9 |  | 1.2 | 28.0 | 40.1 | 30.8 |  | 26.9 | 37.9 | 11.5 | 23.7 | |
|  | Men | 50,811 |  | 15.6 | 50.1 | 30.8 | 3.6 |  | 1.6 | 39.2 | 38.8 | 20.4 |  | 21.7 | 20.5 | 41.6 | 16.2 | |
| **Chile** |  |  |  |  |  |  |  |  |  |  |  |  |  |  |  |  |  | |
| Encuesta de Calidad de Vida y Salud (2016) | Women | 1.364 |  | 10.3 | 52.1 | 33.9 | 3.7 |  | 2.3 | 19.3 | 46.6 | 31.7 |  | 36.0 | 34.5 | 4.5 | 25.0 | |
|  | Men | 2.284 |  | 9.4 | 48.0 | 38.5 | 4.2 |  | 2.2 | 22.2 | 41.1 | 34.5 |  | 34.5 | 15.1 | 34.7 | 15.6 | |
| **Colombia** |  |  |  |  |  |  |  |  |  |  |  |  |  |  |  |  |  | |
| Encuesta Nacional de Calidad de Vida (2017) | Women | 6.705 |  | 14.5 | 53.4 | 28.8 | 3.3 |  | 2.3 | 17.5 | 39.2 | 41.0 |  | 25.9 | 58.4 | 13.1 | 2.7 | |
|  | Men | 10.108 |  | 15.1 | 47.5 | 31.7 | 5.8 |  | 3.2 | 25.6 | 43.5 | 27.7 |  | 15.3 | 25.2 | 32.7 | 26.8 | |
| **Costa Rica** |  |  |  |  |  |  |  |  |  |  |  |  |  |  |  |  |  | |
| II Encuesta Centroamericana sobre Condiciones de Trabajo y Salud (2018) | Women | 497 |  | 10.9 | 51.1 | 34.8 | 3.0 |  | 1.2 | 33.8 | 47.1 | 17.7 |  | 8.9 | 39.4 | 23.7 | 27.8 | |
|  | Men | 1,006 |  | 8.5 | 44.8 | 37.2 | 9.3 |  | 7.1 | 50.5 | 33.2 | 9.2 |  | 8.1 | 12.0 | 48.6 | 31.3 | |
| **Ecuador** |  |  |  |  |  |  |  |  |  |  |  |  |  |  |  |  |  | |
| Encuesta Nacional de Salud y Nutrición (2012) | Women | 12.692 |  | 16.5 | 54.2 | 27.0 | 2.3 |  | 4.2 | 30.7 | 39.4 | 25.6 |  | 15.7 | 42.1 | 19.3 | 22.9 | |
|  | Men | 20.862 |  | 19.6 | 50.2 | 26.8 | 3.4 |  | 2.7 | 36.5 | 44.6 | 16.1 |  | 10.4 | 20.2 | 45.3 | 24.2 | |
| **El Salvador** |  |  |  |  |  |  |  |  |  |  |  |  |  |  |  |  |  | |
| II Encuesta Centroamericana sobre Condiciones de Trabajo y Salud (2018) | Women | 506 |  | 8.7 | 43.1 | 35.6 | 12.6 |  | 8.7 | 38.3 | 42.9 | 9.9 |  | 4.4 | 40.6 | 45.7 | 9.3 | |
|  | Men | 1,001 |  | 11.3 | 37.8 | 30.5 | 20.3 |  | 11.0 | 38.4 | 42.7 | 8.0 |  | 3.6 | 14.5 | 71.9 | 9.9 | |
| **Guatemala** |  |  |  |  |  |  |  |  |  |  |  |  |  |  |  |  |  | |
| II Encuesta Centroamericana sobre Condiciones de Trabajo y Salud (2018) | Women | 881 |  | 42.1 | 36.1 | 17.0 | 4.8 |  | 11.3 | 39.0 | 39.0 | 10.7 |  | 7.2 | 51.3 | 32.8 | 8.8 | |
|  | Men | 629 |  | 28.1 | 36.9 | 27.6 | 7.3 |  | 10.0 | 48.9 | 33.3 | 7.8 |  | 5.7 | 21.8 | 62.4 | 10.1 | |
| **Honduras** |  |  |  |  |  |  |  |  |  |  |  |  |  |  |  |  |  | |
| II Encuesta Centroamericana sobre Condiciones de Trabajo y Salud (2018) | Women | 728 |  | 28.1 | 45.5 | 22.7 | 3.7 |  | 5.3 | 48.9 | 36.6 | 9.2 |  | 6.7 | 35.9 | 36.8 | 20.7 | |
|  | Men | 779 |  | 23.4 | 40.0 | 26.4 | 10.2 |  | 8.7 | 57.4 | 29.2 | 4.8 |  | 6.4 | 11.0 | 64.0 | 18.3 | |
| **México** |  |  |  |  |  |  |  |  |  |  |  |  |  |  |  |  |  | |
| 6ta World Value Survey (2012) | Women | 338 |  | 16.6 | 60.4 | 21.0 | 2.1 |  | 1.5 | 24.6 | 53.0 | 21.0 |  |  |  |  |  | |
|  | Men | 658 |  | 18.1 | 54.6 | 23.1 | 4.3 |  | 2.7 | 20.5 | 55.9 | 20.8 |  |  |  |  |  | |
| **Nicaragua** |  |  |  |  |  |  |  |  |  |  |  |  |  |  |  |  |  | |
| II Encuesta Centroamericana sobre Condiciones de Trabajo y Salud (2018) | Women | 542 |  | 17.2 | 56.5 | 23.2 | 3.0 |  | 7.9 | 32.8 | 40.2 | 19.0 |  | 10.7 | 39.9 | 30.8 | 18.5 | |
|  | Men | 959 |  | 20.4 | 55.0 | 22.1 | 2.5 |  | 11.4 | 37.9 | 36.0 | 14.8 |  | 6.3 | 13.5 | 49.5 | 30.6 | |
| **Panamá** |  |  |  |  |  |  |  |  |  |  |  |  |  |  |  |  |  | |
| II Encuesta Centroamericana sobre Condiciones de Trabajo y Salud (2018) | Women | 502 |  | 15.9 | 52.2 | 28.9 | 2.8 |  | 0.6 | 15.7 | 51.8 | 31.9 |  | 16.9 | 44.6 | 19.7 | 18.5 | |
|  | Men | 1,003 |  | 16.9 | 45.1 | 32.4 | 5.6 |  | 5.4 | 24.6 | 55.8 | 14.1 |  | 7.2 | 13.5 | 58.4 | 20.7 | |
| **Perú** |  |  |  |  |  |  |  |  |  |  |  |  |  |  |  |  |  | |
| I Encuesta de Condiciones de Trabajo y Salud (2016) | Women | 1.442 |  | 22.8 | 47.5 | 23.1 | 6.3 |  | 6.6 | 17.2 | 40.5 | 35.7 |  | 17.9 | 51.1 | 8.2 | 22.9 | |
|  | Men | 1.663 |  | 20.6 | 44.7 | 26.6 | 8.5 |  | 4.3 | 14.6 | 44.2 | 37.5 |  | 21.3 | 29.1 | 36.9 | 12.7 | |
| **Puerto Rico** |  |  |  |  |  |  |  |  |  |  |  |  |  |  |  |  |  | |
| BRFSS Survey Data and Documentation (2017) | Women | 766 |  | 8.1 | 44.5 | 43.5 | 3.9 |  | - | 1.0 | 16.4 | 82.4 |  | - | - | - | - | |
|  | Men | 749 |  | 8.6 | 42.3 | 43.2 | 5.5 |  | 0.1 | 4.0 | 29.1 | 66.8 |  | - | - | - | - | |
| **Uruguay** |  |  |  |  |  |  |  |  |  |  |  |  |  |  |  |  |  | |
| Encuesta Nacional de Salud  (2014) | Women | 842 |  | 12.6 | 49.5 | 35.6 | 2.3 |  | 0.6 | 17.3 | 43.7 | 38.5 |  | 29.0 | 41.2 | 2.9 | 26.9 | |
|  | Men | 849 |  | 14.9 | 47.1 | 34.1 | 3.9 |  | 0.9 | 21.6 | 40.1 | 37.4 |  | 28.1 | 29.0 | 20.3 | 22.5 | |
| **LAC** | Women | 72,683 |  | 16.7 | 49.1 | 30.0 | 4.1 |  | 4.0 | 27.4 | 39.1 | 29.4 |  | 17.1 | 42.3 | 21.9 | 18.6 | |
|  | Men | 107,480 |  | 15.7 | 45.8 | 31.8 | 6.6 |  | 5.4 | 34.0 | 38.1 | 22.5 |  | 13.4 | 17.8 | 48.1 | 20.6 | |
| **Missing values** in education level (EL) and Occupational Categories (OC) : Brazil (EL 3.3%, OC 1.5%); Chile (EL 3.4%, OC 23.3%); Colombia (OC 3.7%); Ecuador (OC 0.6%); Uruguay (OC 0.6%) | | | | | | | | | | | | | | | | | |  |
| **Fuente: Argentina:**Instituto Nacional de Estadística y Censos https://www.indec.gob.ar/indec/web/Institucional-Indec-BasesDeDatos-2 **Brazil**:Instituto Brasileiro de Geografia e Estatística (IBEG) https://ww2.ibge.gov.br/home/estatistica/populacao/pns/2013/default.shtm **Chile**:Departamento de Epidemiología, ministerio de Salud http://epi.minsal.cl/condiciones-de-uso/Colombia:Dirección Nacional de Estadística http://microdatos.dane.gov.co/index.php/catalog/MICRODATOS **Costa Rica; El Salvador; Guatemala; Honduras; Nicaragua; Panma:**University of Texas, U.S.A.; Universidad Naional de Costa Rica: Available under request **Ecuador**:Instituto Nacional de Estadística Censos (INEC) http://www.ecuadorencifras.gob.ec/salud-salud-reproductiva-y-nutricion/ **México**:Institute for Comparative Survey Research http://www.worldvaluessurvey.org/WVSDocumentationWV6.jsp **Perú**:Instituto Nacional de Salud: Available under requestPuerto Rico:Center of Disease Control and Prevention https://www.cdc.gov/brfss/annual_data/annual_2017.html **Uruguay**:Encuesta Nacional de Salud http://www.msp.gub.uy/publicaci%C3%B3n/encuesta-nacional-de-salud | | | | | | | | | | | | | | | | | |  |

| **Supplementary Tabla B. Self-perceived health scale in surveys from 15 countries** | |  |  |  | | | | |  | |  | | | | |  |  | | |  | | |  | |  | |  |
| --- | --- | --- | --- | --- | --- | --- | --- | --- | --- | --- | --- | --- | --- | --- | --- | --- | --- | --- | --- | --- | --- | --- | --- | --- | --- | --- | --- |
| **Country** | **Survey** | **Year** | **Question (original language)** | **Scale** | | | | | | | | | | | | | | | | | | | | **Scale (Original language)** | | |  |
|  |  |  |  | **Good health** | | | | | | | |  | | **Poor Health** | | | | | | | | | |  |  |  |  |
|  |  |  |  | Excellent | | Very good | | Good | | | |  | | Fair | | | | Poor | | | Very poor | | |  |  |  |  |
| Argentina | Encuesta nacional de Factores de Riesgo | 2013 | In general, you would say your health is ….. | | 1 | | 2 | | | 3 | | |  | | 4 | | | | 5 | | |  | | | | 1.Excelente 2. Muy buena 3. Buena 4. Regular 5. Mala | |
| Brazil | National Health Survey | 2013 | In general, how do you consider your health status to be? | |  | | 1 | | | 2 | | |  | | 3 | | | | 4 | | | 5 | | | | 1. Muito boa 2. Boa 3. Regular 4. Ruim 5. Muito ruim | |
| Chile | National Survey of Quality of Life | 2016 | In general, you would say your health is: | | 1 | | 2 | | | 3 | | |  | | 4 | | | | 5 | | |  | | | | 1.Excelente 2. Muy buena 3. Buena 4. Regular 5. Mala | |
| Colombia | National Survey of Quality of Life | 2017 | In general, the health status of "Name" is | |  | | 1 | | | 2 | | |  | | 3 | | | |  | | | 4 | | | | 1. Muy bueno 2. Bueno 3. Regular 4. Muy malo | |
| Costa Rica | II Central American Survey of Working Conditions and Health | 2018 | In general, how do you consider your health status to be? | |  | | 1 | | | 2 | | |  | | 3 | | | | 4 | | | 5 | | | | 1.Muy buena 2. Buena 3. Regular 4. Mala 5. Muy mala | |
| Ecuador | National Health and Nutrition Survey | 2012 | In general, you would say your health is: | | 1 | | 2 | | | 3 | | |  | | 4 | | | | 5 | | |  | | | | 1.Excelente 2. Muy buena 3. Buena 4. Regular 5. Mala | |
| El Salvador | II Central American Survey of Working Conditions and Health | 2018 | In general, how do you consider your health status to be? | |  | | 1 | | | 2 | | |  | | 3 | | | | 4 | | | 5 | | | | 1.Muy buena 2. Buena 3. Regular 4. Mala 5. Muy mala | |
| Guatemala | II Central American Survey of Working Conditions and Health | 2018 | In general, how do you consider your health status to be? | |  | | 1 | | | 2 | | |  | | 3 | | | | 4 | | | 5 | | | | 1.Muy buena 2. Buena 3. Regular 4. Mala 5. Muy mala | |
| Honduras | II Central American Survey of Working Conditions and Health | 2018 | In general, how do you consider your health status to be? | |  | | 1 | | | 2 | | |  | | 3 | | | | 4 | | | 5 | | | | 1.Muy buena 2. Buena 3. Regular 4. Mala 5. Muy mala | |
| México | 6ta World Value Survey | 2012 | In general, how would you describe your health today? | |  | | 1 | | | 2 | | |  | | 3 | | | | 4 | | |  | | | | 1. Muy bueno 2. Bueno 3. Regular 4. Malo | |
| Nicaragua | II Central American Survey of Working Conditions and Health | 2018 | In general, how do you consider your health status to be? | |  | | 1 | | | 2 | | |  | | 3 | | | | 4 | | | 5 | | | | 1.Muy buena 2. Buena 3. Regular 4. Mala 5. Muy mala | |
| Panamá | II Central American Survey of Working Conditions and Health | 2018 | In general, how do you consider your health status to be? | |  | | 1 | | | 2 | | |  | | 3 | | | | 4 | | | 5 | | | | 1.Muy buena 2. Buena 3. Regular 4. Mala 5. Muy mala | |
| Perú | I Encuesta de Condiciones de Trabajo y Salud | 2016 | In general, how do you consider your health status to be? | |  | | 1 | | | 2 | | |  | | 3 | | | | 4 | | | 5 | | | | 1.Muy buena 2. Buena 3. Regular 4. Mala 5. Muy mala | |
| Puerto Rico | BRFSS Survey Data and Documentation | 2017 | In general, you would say your health is: | | 1 | | 2 | | | 3 | | |  | | 4 | | | | 5 | | |  | | | | 1.Excelente 2. Muy buena 3. Buena 4. Regular 5. Mala | |
| Uruguay | National Health Survey | 2014 | In general, you would say your health is: | | 1 | | 2 | | | 3 | | |  | | 4 | | | | 5 | | |  | | | | 1.Excelente 2. Muy buena 3. Buena 4. Regular 5. Mala | |

| \| **Supplementary Table C. Prevalence of poor self-perceived health (%) and 95% confidence interval** \| \| \| \|  \|  \|  \|  \|  \|  \| \| --- \| --- \| --- \| --- \| --- \| --- \| --- \| --- \| --- \| --- \| \|  \|  \|  \|  \|  \|  \|  \|  \|  \|  \| \|  \|  \| **Argentina** \| **Brasil** \| **Chile** \| **Colombia** \| **Costa Rica** \| **Ecuador** \| **El Salvador** \| **Guatemala** \| \|  \|  \| **%(IC95%)** \| **%(IC95%)** \| **%(IC95%)** \| **%(IC95%)** \| **%(IC95%)** \| **%(IC95%)** \| **%(IC95%)** \| **%(IC95%)** \| \| **Women** \| \| 17,9 (17 to 18,9) \| 25.3 (24.8 to 25.7) \| 26,9 (24,5 to 29,2) \| 18,2 (17,3 to 19,1) \| 27,8 (23,8 to 31,7) \| 39,1 (38,3 to 40) \| 31.4 (27.4 to 35.5) \| 19,6 (16,5 to 22,7) \| \| **Age groups** \| \|  \|  \|  \|  \|  \|  \|  \|  \| \|  \| < 25 \| 7,5 (5,9 to 9,1) \| 14.5 (13.7 to 15.4) \| 5 (1,2 to 8,7) \| 10,4 (8,4 to 12,3) \| 11,1 (2,7 to 19,5) \| 26,6 (24,6 to 28,6) \| 11.4 (2 to 20.7) \| 15,5 (11,1 to 19,8) \| \|  \| 25-44 \| 14,1 (12,8 to 15,4) \| 21.1 (20.6 to 21.7) \| 22,7 (19,7 to 25,8) \| 13,7 (12,5 to 14,8) \| 21,3 (16,2 to 26,3) \| 37 (35,8 to 38,1) \| 26.1 (20.3 to 32) \| 15,4 (10,7 to 20,1) \| \|  \| 45-64 \| 26,7 (24,7 to 28,8) \| 36.7 (35.9 to 37.6) \| 37,9 (33,5 to 42,3) \| 27,6 (25,6 to 29,6) \| 41 (33,7 to 48,4) \| 48,3 (46,6 to 50) \| 35.6 (28.6 to 42.5) \| 33,6 (24,7 to 42,6) \| \|  \| >65 \| 29,8 (29,7 to 39,8) \| 45.3 (41.7 to 48.8) \| 35,6 (25,7 to 52,4) \| 35,2 (35,5 to 48,4) \| 46,7 (21,4 to 71,9) \| 53,4 (55,8 to 67) \| 51.6 (39.3 to 63.8) \| 36,7 (19,4 to 53,9) \| \| **Educational level** \| \|  \|  \|  \|  \|  \|  \|  \|  \| \|  \| High \| 9,4 (8,1 to 10,6) \| 12.9 (12.2 to 13.5) \| 19,3 (15,5 to 23,1) \| 10,3 (9,1 to 11,4) \| 20,5 (53,5 to 113,2) \| 26,1 (24,6 to 27,6) \| 24 (39.8 to 69.3) \| 11,9 (25,4 to 47,8) \| \|  \| Middle \| 18 (16 to 19,9) \| 21.9 (21.3 to 22.6) \| 26,8 (23,3 to 30,3) \| 18,5 (17 to 20) \| 25,6 (25,6 to 39,8) \| 39,8 (38,4 to 41,2) \| 24.9 (28.8 to 42.3) \| 17,1 (13,5 to 23,2) \| \|  \| Low \| 23,4 (21,5 to 25,2) \| 40.2 (39.2 to 41.1) \| 36,7 (30,7 to 42,6) \| 34,8 (32,1 to 37,5) \| 32,7 (20 to 31,2) \| 47,4 (45,9 to 49) \| 35.6 (19.1 to 30.6) \| 18,4 (12,4 to 21,9) \| \|  \| >Low \| 34,2 (29,6 to 38,7) \| 64.4 (59.9 to 68.8) \| 34,6 (17,8 to 51,5) \| 28,8 (21,7 to 36) \| 83,3 (12 to 28,9) \| 52,1 (47,9 to 56,2) \| 54.5 (12.2 to 35.8) \| 36,6 (4,2 to 19,7) \| \| **Occupational categories** \| \|  \|  \|  \|  \|  \|  \|  \|  \| \|  \| Non-manual skilled \| - \| 14.5 (13.8 to 15.2) \| 20 (15,9 to 24,1) \| 10,6 (9,2 to 12,1) \| 13,6 (3,5 to 23,8) \| 25,8 (23,9 to 27,7) \| 36.4 (16.3 to 56.5) \| 13,3 (3,4 to 23,3) \| \|  \| Non-nanual non-skilled \| - \| 23.0 (22.3 to 23.7) \| 29 (24,2 to 33,8) \| 20,1 (18,9 to 21,4) \| 28,6 (22,2 to 34,9) \| 39,7 (38,4 to 41) \| 26.8 (20.8 to 32.9) \| 16,1 (12,1 to 20,2) \| \|  \| Manual skilled \| - \| 36.1 (34.7 to 37.5) \| 29,4 (16,1 to 42,7) \| 28,3 (25,2 to 31,3) \| 32,2 (23,8 to 40,6) \| 42,7 (40,8 to 44,7) \| 33.3 (27.3 to 39.4) \| 27,2 (21,1 to 33,3) \| \|  \| Manual non-skilled \| - \| 36.4 (35.4 to 37.4) \| 31,4 (25,6 to 37,1) \| 19,1 (13,3 to 25) \| 27,5 (20,1 to 35) \| 44,5 (42,7 to 46,3) \| 40.4 (26.4 to 54.5) \| 12,7 (3,9 to 21,5) \| \| **Men** \| \| 14,1 (13,5 to 14,7) \| 22.3 (21.9 to 22.6) \| 14,5 (13,1 to 15,9) \| 13,4 (12,8 to 14,1) \| 25,5 (22,9 to 28,2) \| 33,7 (33 to 34,3) \| 26.8 (24 to 29.5) \| 24,3 (21,5 to 27,1) \| \| **Age groups** \| \|  \|  \|  \|  \|  \|  \|  \|  \| \|  \| < 25 \| 5,6 (4,5 to 6,6) \| 11.6 (10.9 to 12.3) \| 7 (3,4 to 10,5) \| 5,6 (4,4 to 6,8) \| 16,3 (8,5 to 24,1) \| 22,8 (21,5 to 24,2) \| 11.5 (5.6 to 17.4) \| 18,5 (13,7 to 23,4) \| \|  \| 25-44 \| 10,4 (9,7 to 11,1) \| 17.5 (17 to 18) \| 9,7 (8 to 11,5) \| 9,3 (8,5 to 10,2) \| 16,6 (13,2 to 20,1) \| 31,3 (30,4 to 32,1) \| 21.2 (17 to 25.3) \| 17,5 (13,4 to 21,7) \| \|  \| 45-64 \| 21,2 (20 to 22,4) \| 33 (32.3 to 33.8) \| 19,8 (17,2 to 22,5) \| 18,9 (17,5 to 20,2) \| 35,8 (31 to 40,7) \| 42,5 (41,2 to 43,8) \| 26.2 (21.3 to 31.2) \| 32,5 (26,6 to 38,4) \| \|  \| >65 \| 29,8 (26,4 to 33,2) \| 43.4 (41.1 to 45.7) \| 35,6 (26,1 to 45,2) \| 35,2 (31,4 to 39,1) \| 35,1 (25,5 to 44,8) \| 53,4 (49,8 to 57) \| 45.8 (39 to 52.7) \| 50 (37,8 to 62,3) \| \| **Educational level** \| \|  \|  \|  \|  \|  \|  \|  \|  \| \|  \| High \| 6,5 (5,8 to 7,3) \| 10.5 (9.9 to 11.1) \| 6 (4,3 to 7,7) \| 5,8 (4,9 to 6,6) \| 9,7 (52,2 to 74,6) \| 20,3 (19 to 21,6) \| 10 (28.2 to 46.3) \| 7,2 (14 to 31,5) \| \|  \| Middle \| 11,2 (10,2 to 12,1) \| 16.3 (15.7 to 16.8) \| 15,7 (13,3 to 18,1) \| 10,7 (9,8 to 11,6) \| 18,6 (23,9 to 31,6) \| 31,5 (30,5 to 32,5) \| 23.2 (26.6 to 35.9) \| 16 (28,5 to 37,4) \| \|  \| Low \| 19,8 (18,7 to 20,9) \| 31.1 (30.5 to 31.8) \| 23,4 (19,7 to 27,2) \| 25,1 (23,4 to 26,8) \| 27,8 (14,4 to 22,7) \| 42,2 (41,1 to 43,3) \| 31.3 (19.2 to 27.2) \| 32,9 (11,8 to 20,2) \| \|  \| >Low \| 32,2 (29,1 to 35,2) \| 48 (44.5 to 51.4) \| 13,9 (4,2 to 23,5) \| 24,1 (19,5 to 28,6) \| 63,4 (3,7 to 15,7) \| 52,7 (48,6 to 56,8) \| 37.3 (3.4 to 16.6) \| 22,7 (1,1 to 13,4) \| \| **Occupational categories** \| \|  \|  \|  \|  \|  \|  \|  \|  \| \|  \| Non-manual skilled \| - \| 12.7 (12.1 to 13.3) \| 8,1 (6 to 10,3) \| 6,3 (5 to 7,5) \| 13,6 (6,1 to 21) \| 20,5 (18,9 to 22,2) \| 11.1 (0.8 to 21.4) \| 6 (-0,6 to 12,6) \| \|  \| Non-nanual non-skilled \| - \| 20.3 (19.5 to 21.1) \| 7,6 (4,4 to 10,8) \| 11,3 (10,1 to 12,6) \| 17,4 (10,6 to 24,1) \| 29,5 (28,1 to 30,8) \| 32.4 (24.8 to 40) \| 13 (8,3 to 17,8) \| \|  \| Manual skilled \| - \| 25.7 (25.1 to 26.3) \| 19,7 (16,6 to 22,8) \| 19,9 (18,5 to 21,3) \| 33,7 (29,6 to 37,9) \| 36 (35 to 36,9) \| 25.6 (22.4 to 28.7) \| 30 (26,2 to 33,8) \| \|  \| Manual non-skilled \| - \| 30.3 (29.3 to 31.3) \| 23,1 (18,2 to 28,1) \| 13,5 (12,2 to 14,8) \| 19 (14,7 to 23,4) \| 39,6 (38,2 to 40,9) \| 32.3 (23.1 to 41.5) \| 23,6 (14,8 to 32,4) \| \|  \|  \|  \|  \|  \|  \|  \|  \|  \|  \| \|  \|  \|  \| **Honduras** \| **Mexico** \| **Nicaragua** \| **Panama** \| **Peru** \| **Puerto Rico** \| **Uruguay** \| \|  \|  \|  \| **%(IC95%)** \| **%(IC95%)** \| **%(IC95%)** \| **%(IC95%)** \| **%(IC95%)** \| **%(IC95%)** \| **%(IC95%)** \| \| **Women** \| \|  \| 43,2 (39,3 to 47) \| 24.0 (19,4 to 28,5) \| 48.0 (43,8 to 52,2) \| 30,1 (26,1 to 34,1) \| 43,8 (41,3 to 46,4) \| 24,5 (21,5 to 27,6) \| 15,2 (12,8 to 17,6) \| \| **Age groups** \| \|  \|  \|  \|  \|  \|  \|  \|  \| \|  \| < 25 \|  \| 35,4 (28,4 to 42,3) \| 10,7 (2,6 to 18,8) \| 35,5 (25,8 to 45,2) \| 13,8 (6,2 to 21,3) \| 29,3 (24,4 to 34,2) \| 6,5 (0,3 to 12,6) \| 4,8 (0,7 to 8,9) \| \|  \| 25-44 \|  \| 38,6 (33 to 44,1) \| 20,6 (15 to 26,1) \| 45,1 (39,5 to 50,7) \| 27,9 (22,4 to 33,3) \| 39 (35,3 to 42,6) \| 20,5 (16,2 to 24,8) \| 14,4 (11,1 to 17,8) \| \|  \| 45-64 \|  \| 56,2 (48,1 to 64,2) \| 38 (26,7 to 49,3) \| 60,3 (51,8 to 68,9) \| 38,6 (30,7 to 46,5) \| 55,1 (49,7 to 60,4) \| 30,3 (25,4 to 35,3) \| 19,5 (15 to 24) \| \|  \| >65 \|  \| 79,2 (62,9 to 95,4) \| 46,4 (59,8 to 111,6) \| 75 (53,8 to 96,2) \| 71,4 (47,8 to 95,1) \| 55,5 (81,1 to 94,5) \| 31,7 (25,6 to 61,1) \| 10,6 (3,3 to 40,1) \| \| **Educational level** \| \|  \|  \|  \|  \|  \|  \|  \|  \| \|  \| High \|  \| 20,3 (42,3 to 75,4) \| 9,9 (2,9 to 16,8) \| 33.0 (50,9 to 79,4) \| 25,6 (13,3 to 120) \| 32,1 (28,1 to 36,2) \| 21,9 (18,6 to 25,1) \| 9,3 (6,1 to 12,5) \| \|  \| Middle \|  \| 30,9 (49,4 to 60,4) \| 19,6 (13,7 to 25,4) \| 44 (50 to 64,6) \| 25 (43,4 to 65,4) \| 41,6 (37,6 to 45,6) \| 34,9 (26,6 to 43,2) \| 15,9 (12,2 to 19,7) \| \|  \| Low \|  \| 54,9 (25 to 36,8) \| 42,2 (31,5 to 52,8) \| 57,3 (37,4 to 50,6) \| 54,4 (19,7 to 30,3) \| 59,4 (53,3 to 65,5) \| 62,5 (29 to 96) \| 26,1 (18,9 to 33,2) \| \|  \| >Low \|  \| 58,8 (10,1 to 30,6) \| 80 (44,9 to 115,1) \| 65,1 (23,9 to 42,1) \| 66,7 (18,9 to 32,4) \| 77,9 (69,5 to 86,2) \| - \| 26,9 (-12,4 to 66,3) \| \| **Occupational categories** \| \|  \|  \|  \|  \|  \|  \|  \|  \| \|  \| Non-manual skilled \|  \| 37,2 (22,8 to 51,7) \| 6,7 (-0,6 to 14) \| 37,9 (25,4 to 50,4) \| 24,7 (15,5 to 33,9) \| 28,1 (22,6 to 33,6) \| - \| 9,7 (6 to 13,4) \| \|  \| Non-nanual non-skilled \|  \| 42,9 (36,5 to 49,2) \| 16,3 (5,2 to 27,3) \| 44,9 (38,3 to 51,5) \| 23,7 (18,1 to 29,2) \| 41,5 (37,9 to 45) \| - \| 12,8 (9,3 to 16,3) \| \|  \| Manual skilled \|  \| 43,9 (37,6 to 50,2) \| 19 (10,7 to 27,4) \| 59,9 (52,4 to 67,3) \| 43,4 (33,7 to 53,2) \| 52,3 (43,3 to 61,3) \| - \| 15 (0,8 to 29,2) \| \|  \| Manual non-skilled \|  \| 44,4 (35,9 to 52,8) \| 33,1 (25,9 to 40,4) \| 40 (30,4 to 49,6) \| 35,5 (25,8 to 45,2) \| 57,8 (52,5 to 63,1) \| - \| 24,8 (19,2 to 30,4) \| \| **Men** \| \|  \| 43,8 (40,5 to 47,1) \| 22,9 (19,7 to 26,2) \| 43,4 (40,2 to 46,5) \| 22,9 (20,3 to 25,5) \| 34,4 (32,1 to 36,7) \| 18,2 (15,4 to 20,9) \| 9,2 (7,3 to 11,1) \| \| **Age groups** \| \|  \|  \|  \|  \|  \|  \|  \|  \| \|  \| < 25 \|  \| 18,8 (13,4 to 24,2) \| 18,5 (11,5 to 25,5) \| 27,6 (21,3 to 33,8) \| 11,8 (6,9 to 16,6) \| 20,6 (16,3 to 24,9) \| 7,7 (1,2 to 14,2) \| 7,4 (2,8 to 11,9) \| \|  \| 25-44 \|  \| 38,8 (33,7 to 44) \| 17 (13,1 to 20,9) \| 41,7 (37,5 to 46) \| 16,4 (13 to 19,8) \| 29,7 (26,4 to 33) \| 12,6 (8,9 to 16,2) \| 6,4 (4 to 8,8) \| \|  \| 45-64 \|  \| 61,4 (55,1 to 67,7) \| 36,2 (28,5 to 43,8) \| 59 (52,3 to 65,6) \| 32 (26,9 to 37,1) \| 46,4 (41,7 to 51) \| 24 (19,4 to 28,6) \| 13,6 (9,7 to 17,6) \| \|  \| >65 \|  \| 75 (66 to 84) \| 46,4 (28 to 64,9) \| 70,8 (52,6 to 89) \| 57,1 (44,2 to 70,1) \| 55,5 (47,3 to 63,7) \| 31,7 (17,5 to 46) \| 10,6 (0,1 to 21,1) \| \| **Educational level** \| \|  \|  \|  \|  \|  \|  \|  \|  \| \|  \| High \|  \| 26,8 (51,7 to 73,6) \| 10,9 (5,7 to 16,2) \| 31,7 (47,6 to 66,2) \| 16,3 (27,6 to 53,8) \| 26 (22,5 to 29,4) \| 12,5 (9,7 to 15,4) \| 6,9 (4,1 to 9,7) \| \|  \| Middle \|  \| 24,2 (47,9 to 56,7) \| 20,7 (16,5 to 24,8) \| 37,1 (44,7 to 55) \| 16,1 (32 to 44,1) \| 34,2 (30,8 to 37,7) \| 27,9 (21,9 to 33,8) \| 8,5 (5,6 to 11,5) \| \|  \| Low \|  \| 52,3 (18,9 to 29,5) \| 38,5 (30,3 to 46,7) \| 49,9 (32 to 42,2) \| 38,1 (13 to 19,1) \| 47,2 (40,9 to 53,5) \| 36,7 (19,4 to 53,9) \| 13,3 (8,4 to 18,2) \| \|  \| >Low \|  \| 62,7 (13,3 to 40,4) \| 44,4 (21,5 to 67,4) \| 56,9 (24 to 39,3) \| 40,7 (10,2 to 22,4) \| 64 (52,9 to 75,2) \| - \| 34 (1 to 67) \| \| **Occupational categories** \| \|  \|  \|  \|  \|  \|  \|  \|  \| \|  \| Non-manual skilled \|  \| 23,6 (12,4 to 34,9) \| 18,2 (10,1 to 26,2) \| 30 (18,4 to 41,6) \| 16,7 (8,1 to 25,3) \| 24,7 (20,2 to 29,2) \| - \| 5,8 (2,9 to 8,8) \| \|  \| Non-nanual non-skilled \|  \| 33,7 (24,2 to 43,2) \| 16,2 (7,4 to 24,9) \| 36,4 (28,1 to 44,7) \| 25,2 (17,9 to 32,5) \| 29,6 (25,6 to 33,7) \| - \| 8,4 (5 to 11,9) \| \|  \| Manual skilled \|  \| 47,1 (42,9 to 51,3) \| 28,4 (21,9 to 34,9) \| 45,3 (40,8 to 49,7) \| 24,1 (20,6 to 27,5) \| 40,9 (37 to 44,8) \| - \| 7,5 (3,6 to 11,4) \| \|  \| Manual non-skilled \|  \| 45,6 (37,8 to 53,3) \| 22,5 (17,9 to 27,2) \| 46,1 (40,4 to 51,8) \| 20,2 (14,7 to 25,6) \| 42,8 (36,1 to 49,5) \| - \| 16,1 (10,9 to 21,4) \| |  |  |  |  |  |  |
| --- | --- | --- | --- | --- | --- | --- | --- | --- | --- | --- | --- | --- | --- | --- | --- | --- | --- | --- | --- | --- | --- | --- | --- | --- | --- | --- | --- | --- | --- | --- | --- | --- | --- | --- | --- | --- | --- | --- | --- | --- | --- | --- | --- | --- | --- | --- | --- | --- | --- | --- | --- | --- | --- | --- | --- | --- | --- | --- | --- | --- | --- | --- | --- | --- | --- | --- | --- | --- | --- | --- | --- | --- | --- | --- | --- | --- | --- | --- | --- | --- | --- | --- | --- | --- | --- | --- | --- | --- | --- | --- | --- | --- | --- | --- | --- | --- | --- | --- | --- | --- | --- | --- | --- | --- | --- | --- | --- | --- | --- | --- | --- | --- | --- | --- | --- | --- | --- | --- | --- | --- | --- | --- | --- | --- | --- | --- | --- | --- | --- | --- | --- | --- | --- | --- | --- | --- | --- | --- | --- | --- | --- | --- | --- | --- | --- | --- | --- | --- | --- | --- | --- | --- | --- | --- | --- | --- | --- | --- | --- | --- | --- | --- | --- | --- | --- | --- | --- | --- | --- | --- | --- | --- | --- | --- | --- | --- | --- | --- | --- | --- | --- | --- | --- | --- | --- | --- | --- | --- | --- | --- | --- | --- | --- | --- | --- | --- | --- | --- | --- | --- | --- | --- | --- | --- | --- | --- | --- | --- | --- | --- | --- | --- | --- | --- | --- | --- | --- | --- | --- | --- | --- | --- | --- | --- | --- | --- | --- | --- | --- | --- | --- | --- | --- | --- | --- | --- | --- | --- | --- | --- | --- | --- | --- | --- | --- | --- | --- | --- | --- | --- | --- | --- | --- | --- | --- | --- | --- | --- | --- | --- | --- | --- | --- | --- | --- | --- | --- | --- | --- | --- | --- | --- | --- | --- | --- | --- | --- | --- | --- | --- | --- | --- | --- | --- | --- | --- | --- | --- | --- | --- | --- | --- | --- | --- | --- | --- | --- | --- | --- | --- | --- | --- | --- | --- | --- | --- | --- | --- | --- | --- | --- | --- | --- | --- | --- | --- | --- | --- | --- | --- | --- | --- | --- | --- | --- | --- | --- | --- | --- | --- | --- | --- | --- | --- | --- | --- | --- | --- | --- | --- | --- | --- | --- | --- | --- | --- | --- | --- | --- | --- | --- | --- | --- | --- | --- | --- | --- | --- | --- | --- | --- | --- | --- | --- | --- | --- | --- | --- | --- | --- | --- | --- | --- | --- | --- | --- | --- | --- | --- | --- | --- | --- | --- | --- | --- | --- | --- | --- | --- | --- | --- | --- | --- | --- | --- | --- | --- | --- | --- | --- | --- | --- | --- | --- | --- | --- | --- | --- | --- | --- | --- | --- | --- | --- | --- | --- | --- | --- | --- | --- | --- | --- | --- | --- | --- | --- | --- | --- | --- | --- | --- | --- | --- | --- | --- | --- | --- | --- | --- | --- | --- | --- | --- | --- | --- | --- | --- | --- | --- | --- | --- | --- | --- | --- | --- | --- | --- | --- | --- | --- | --- | --- | --- | --- | --- | --- | --- | --- | --- | --- | --- | --- | --- | --- | --- | --- | --- | --- | --- | --- | --- | --- | --- | --- | --- | --- | --- | --- | --- | --- | --- | --- | --- | --- | --- | --- | --- | --- | --- | --- | --- | --- | --- | --- | --- | --- | --- | --- | --- | --- | --- | --- | --- | --- | --- | --- | --- | --- | --- | --- | --- | --- | --- | --- | --- | --- | --- | --- | --- | --- | --- | --- | --- | --- | --- | --- | --- | --- | --- | --- | --- | --- | --- | --- | --- | --- | --- | --- | --- | --- | --- | --- | --- | --- | --- | --- | --- | --- | --- | --- | --- | --- | --- | --- | --- | --- | --- | --- | --- | --- | --- | --- | --- | --- | --- | --- | --- | --- | --- | --- | --- | --- | --- | --- | --- | --- | --- | --- | --- | --- | --- | --- | --- | --- | --- | --- | --- | --- | --- | --- | --- | --- | --- | --- | --- | --- | --- | --- | --- | --- | --- | --- | --- | --- | --- | --- | --- | --- | --- | --- | --- | --- | --- | --- | --- | --- | --- | --- | --- | --- | --- | --- | --- | --- | --- | --- | --- | --- | --- | --- | --- | --- | --- | --- | --- | --- | --- | --- | --- | --- | --- | --- | --- | --- | --- | --- | --- | --- | --- | --- | --- | --- | --- | --- | --- | --- | --- | --- | --- | --- | --- | --- | --- | --- | --- | --- | --- | --- | --- | --- | --- | --- | --- | --- | --- | --- | --- | --- | --- | --- | --- | --- | --- | --- | --- | --- | --- | --- | --- | --- | --- | --- | --- | --- | --- | --- | --- | --- | --- | --- | --- | --- | --- | --- | --- | --- |

| \| **Supplementary table D. Prevalence of poor self-perceived health (%) and Kuznets relative index with 95% confidence interval** \| \| \| \| \| \| \| \| \| \| \| \| \| \| \| \| \| \| \| --- \| --- \| --- \| --- \| --- \| --- \| --- \| --- \| --- \| --- \| --- \| --- \| --- \| --- \| --- \| --- \| --- \| --- \| \|  \|  \|  \|  \|  \|  \|  \|  \|  \|  \|  \|  \|  \|  \|  \|  \|  \|  \| \|  \|  \| **Argentina** \| \| **Brasil** \| \| **Chile** \| \| **Colombia** \| \| **Costa Rica** \| \| **Ecuador** \| \| **El Salvador** \| \| **Guatemala** \| \| \|  \|  \| **%** \| **Kzt (CI 95%)** \| **%** \| **Kzt (CI 95%)** \| **%** \| **Kzt (CI 95%)** \| **%** \| **Kzt (CI 95%)** \| **%** \| **Kzt (CI 95%)** \| **%** \| **Kzt (CI 95%)** \| **%** \| **Kzt (CI 95%)** \| **%** \| **Kzt (CI 95%)** \| \| **Women** \| \| 17.9 \|  \| 25.0 \|  \| 26.9 \|  \| 18.2 \|  \| 27.8 \|  \| 39.1 \|  \| 31.4 \|  \| 19.6 \|  \| \| **Age groups** \| \|  \|  \|  \|  \|  \|  \|  \|  \|  \|  \|  \|  \|  \|  \|  \|  \| \|  \| < 25 \| 7.5 \| **1** \| 14.5 \| **1** \| 4.5 \| **1** \| 12.2 \| **1** \| 11.1 \| **1** \| 27.1 \| **1** \| 11.4 \| **1** \| 15.5 \| **1** \| \|  \| 25-44 \| 14.1 \| 1,9 (1,5 - 2,4) \| 21.1 \| 1.5 (1.4 - 1.6) \| 22.7 \| 4,6 (2,1 - 9,9) \| 15.2 \| 1,3 (1,1 - 1,6) \| 21.3 \| 1.9 (0.9 - 4.2) \| 37.0 \| 1,4 (1,3 - 1,5) \| 26.1 \| 2.3 (1 - 5.4) \| 15.4 \| 1 (0.7 - 1.5) \| \|  \| 45-64 \| 26.7 \| 3,6 (2,8 - 4,5) \| 36.7 \| 2.5 (2.4 - 2.7) \| 37.9 \| 7,6 (3,6 - 16,4) \| 29.6 \| 2,7 (2,2 - 3,3) \| 41.0 \| 3.7 (1.7 - 8) \| 48.3 \| 1,8 (1,7 - 2) \| 35.6 \| 3.1 (1.3 - 7.3) \| 33.6 \| 2.2 (1.5 - 3.2) \| \|  \| >65 \| 34.8 \| 4,6 (3,6 - 6) \| 45.3 \| 3.1 (2.8 - 3.4) \| 39.1 \| 7,9 (3,4 - 18,1) \| 49.4 \| 4 (3,2 - 5,2) \| 46.7 \| 4.2 (1.7 - 10.6) \| 61.4 \| 2,3 (2 - 2,6) \| 51.6 \| 4.5 (1.9 - 10.7) \| 36.7 \| 2.4 (1.4 - 4.1) \| \| **Educational level** \| \|  \|  \|  \|  \|  \|  \|  \|  \|  \|  \|  \|  \|  \|  \|  \|  \| \|  \| High \| 9.4 \| **1** \| 12.8 \| **1** \| 19.3 \| **1** \| 11.5 \| **1** \| 20.5 \| **1** \| 26.1 \| **1** \| 24.0 \| **1** \| 11.9 \| **1** \| \|  \| Middle \| 18.0 \| 1,7 (1,5 - 2,1) \| 21.5 \| 1.7 (1.6 - 1.8) \| 26.8 \| 1.5 (1.4 - 1.6) \| 19.4 \| 1,8 (1,6 - 2,1) \| 25.6 \| 1.3 (0.8 - 2) \| 39.3 \| 1,5 (1,4 - 1,6) \| 24.9 \| 1 (0.6 - 1.8) \| 17.1 \| 1.4 (0.7 - 2.9) \| \|  \| Low \| 23.4 \| 2,8 (2,4 - 3,3) \| 39.8 \| 3.1 (3.0 - 3.3) \| 36.7 \| 2.9 (2.8 - 3.1) \| 37.4 \| 3,4 (3 - 3,9) \| 32.7 \| 1.6 (1 - 2.5) \| 47.4 \| 1,8 (1,7 - 1,9) \| 35.6 \| 1.5 (0.9 - 2.5) \| 18.4 \| 1.5 (0.8 - 3.1) \| \|  \| >Low \| 34.2 \| 4,4 (3,6 - 5,3) \| 63.6 \| 5 (4.6 - 5.4) \| 34.6 \| 4.6 (4.2 - 5) \| 38.7 \| 2,8 (2,1 - 3,7) \| 83.3 \| 4.1 (2.4 - 7) \| 52.0 \| 2 (1,8 - 2,2) \| 54.5 \| 2.3 (1.3 - 4) \| 36.6 \| 3.1 (1.5 - 6.3) \| \| **Occupational categories** \| \| \|  \|  \|  \|  \|  \|  \|  \|  \|  \|  \|  \|  \|  \|  \|  \| \|  \| Non-manual skilled \| - \| - \| 14.5 \| **1** \| 20.0 \| **1** \| 11.9 \| **1** \| 13.6 \| **1** \| 25.8 \| **1** \| 36.4 \| **1** \| 13.3 \| **1** \| \|  \| Non-nanual non-skilled \| - \| - \| 22.6 \| 1.6 (1.5 - 1.7) \| 29.0 \| 1,5 (1,1 - 1,9) \| 22.6 \| 1,9 (1,6 - 2,2) \| 28.6 \| 2.1 (1 - 4.6) \| 39.8 \| 1,5 (1,4 - 1,7) \| 26.8 \| 0.7 (0.4 - 1.3) \| 16.1 \| 1.2 (0.6 - 2.7) \| \|  \| Manual skilled \| - \| - \| 36.0 \| 2.5 (2.3 - 2.6) \| 29.4 \| 1,5 (0,9 - 2,4) \| 30.9 \| 2,7 (2,2 - 3,2) \| 32.2 \| 2.4 (1.1 - 5.2) \| 42.3 \| 1,6 (1,5 - 1,8) \| 33.3 \| 0.9 (0.5 - 1.6) \| 27.2 \| 2 (0.9 - 4.4) \| \|  \| Manual non-skilled \| - \| - \| 36.0 \| 2.5 (2.4 - 2.7) \| 31.4 \| 1,6 (1,2 - 2,1) \| 24.3 \| 1,8 (1,3 - 2,5) \| 27.5 \| 2 (0.9 - 4.5) \| 43.7 \| 1,7 (1,6 - 1,8) \| 40.4 \| 1.1 (0.6 - 2.1) \| 12.7 \| 1 (0.3 - 2.6) \| \| **Men** \| \| 14.1 \|  \| 22.0 \|  \| 14.5 \|  \| 13.4 \|  \| 25.5 \|  \| 33.7 \|  \| 26.8 \|  \| 24.3 \|  \| \| **Age groups** \| \|  \|  \|  \|  \|  \|  \|  \|  \|  \|  \|  \|  \|  \|  \|  \|  \| \|  \| < 25 \| 6.7 \| **1** \| 11.5 \| **1** \| 6.4 \| **1** \| 6.1 \| **1** \| 16.3 \| **1** \| 22.4 \| **1** \| 11.5 \| **1** \| 18.5 \| **1** \| \|  \| 25-44 \| 10.4 \| 1,9 (1,5 - 2,3) \| 17.5 \| 1.5 (1.4 - 1.6) \| 9.7 \| 1,4 (0,8 - 2,4) \| 10.6 \| 1,7 (1,3 - 2,1) \| 16.6 \| 1 (0.6 - 1.7) \| 31.3 \| 1,4 (1,3 - 1,5) \| 21.2 \| 1.8 (1.1 - 3.2) \| 17.5 \| 0.9 (0.7 - 1.3) \| \|  \| 45-64 \| 22.0 \| 3,8 (3,1 - 4,6) \| 33.0 \| 2.8 (2.7 - 3) \| 19.8 \| 2,8 (1,7 - 4,8) \| 21.5 \| 3,4 (2,7 - 4,3) \| 35.8 \| 2.2 (1.3 - 3.6) \| 42.5 \| 1,9 (1,8 - 2) \| 26.2 \| 2.3 (1.3 - 3.9) \| 32.5 \| 1.8 (1.3 - 2.4) \| \|  \| >65 \| 28.4 \| 5,4 (4,3 - 6,7) \| 43.4 \| 3.7 (3.5 - 4.1) \| 35.6 \| 5,1 (2,9 - 9,1) \| 38.9 \| 6,3 (4,9 - 8,1) \| 35.1 \| 2.2 (1.2 - 3.7) \| 53.4 \| 2,4 (2,2 - 2,6) \| 45.8 \| 4 (2.3 - 6.8) \| 50.0 \| 2.7 (1.9 - 3.9) \| \| **Educational level** \| \|  \|  \|  \|  \|  \|  \|  \|  \|  \|  \|  \|  \|  \|  \|  \|  \| \|  \| High \| 6.9 \| **1** \| 10.4 \| **1** \| 6.0 \| **1** \| 7.0 \| **1** \| 9.7 \| **1** \| 20.3 \| **1** \| 10.0 \| **1** \| 7.2 \| **1** \| \|  \| Middle \| 11.3 \| 1,7 (1,5 - 2) \| 15.9 \| 1.6 (1.5 - 1.7) \| 15.7 \| 2,3 (1,1 - 4,9) \| 12.1 \| 1,8 (1,6 - 2,2) \| 18.6 \| 1.9 (1 - 3.7) \| 29.5 \| 1,5 (1,4 - 1,6) \| 23.2 \| 2.3 (1.2 - 4.6) \| 16.0 \| 2.2 (0.9 - 5.4) \| \|  \| Low \| 20.4 \| 3 (2,7 - 3,4) \| 30.7 \| 3.0 (2.8 - 3.2) \| 23.4 \| 3,9 (2,8 - 5,4) \| 26.1 \| 4,3 (3,7 - 5,1) \| 27.8 \| 2.9 (1.5 - 5.4) \| 42.2 \| 2,1 (1,9 - 2,2) \| 31.3 \| 3.1 (1.6 - 6.1) \| 32.9 \| 4.5 (1.9 - 10.7) \| \|  \| >Low \| 30.6 \| 4,9 (4,2 - 5,7) \| 47.7 \| 4.6 (4.2 - 5.0) \| 13.9 \| 2,3 (1,1 - 4,9) \| 28.9 \| 4,2 (3,3 - 5,3) \| 63.4 \| 6.5 (3.4 - 12.5) \| 52.5 \| 2,6 (2,3 - 2,9) \| 37.3 \| 3.7 (1.8 - 7.5) \| 22.7 \| 3.1 (1.2 - 7.9) \| \| **Occupational categories** \| \| \|  \|  \|  \|  \|  \|  \|  \|  \|  \|  \|  \|  \|  \|  \|  \| \|  \| Non-manual skilled \| - \| - \| 12.6 \| **1** \| 8.1 \| **1** \| 8.0 \| **1** \| 13.6 \| **1** \| 20.5 \| **1** \| 11.1 \| **1** \| 6.0 \| **1** \| \|  \| Non-nanual non-skilled \| - \| - \| 20.0 \| 1.6 (1.5 - 1.7) \| 7.6 \| 0,9 (0,6 - 1,5) \| 13.8 \| 1,8 (1,4 - 2,3) \| 17.4 \| 1.3 (0.7 - 2.5) \| 29.6 \| 1,4 (1,3 - 1,6) \| 32.4 \| 2.9 (1.1 - 7.6) \| 13.0 \| 2.2 (0.7 - 6.9) \| \|  \| Manual skilled \| - \| - \| 25.4 \| 2.0 (1.9 - 2.1) \| 19.7 \| 2,4 (1,8 - 3,3) \| 21.0 \| 3,2 (2,6 - 3,9) \| 33.7 \| 2.5 (1.4 - 4.4) \| 35.6 \| 1,7 (1,6 - 1,9) \| 25.6 \| 2.3 (0.9 - 5.8) \| 30.0 \| 5 (1.7 - 15.1) \| \|  \| Manual non-skilled \| - \| - \| 29.3 \| 2.4 (2.3 - 2.5) \| 23.1 \| 2,8 (2 - 4) \| 14.7 \| 2,2 (1,7 - 2,7) \| 19.0 \| 1.4 (0.8 - 2.5) \| 38.3 \| 1,9 (1,7 - 2) \| 32.3 \| 2.9 (1.1 - 7.7) \| 23.6 \| 3.9 (1.2 - 12.5) \| \|  \|  \|  \|  \|  \|  \|  \|  \|  \|  \|  \|  \|  \|  \|  \|  \|  \|  \| \| **Continue… Supplementary Table D. Prevalence of poor self-perceived health (%) and Kuznets relative index with 95% confidence interval** \| \| \| \| \| \| \| \| \| \|  \|  \|  \|  \|  \|  \|  \|  \| \|  \|  \|  \|  \|  \|  \|  \|  \|  \|  \|  \|  \|  \|  \|  \|  \|  \|  \| \|  \|  \|  \| \| **Mexico** \| \| **Honduras** \| \| **Nicaragua** \| \| **Panama** \| \| **Peru** \| \| **Puerto Rico** \| \| **Uruguay** \|  \| \|  \|  \|  \|  \| **%** \| **Kzt (CI 95%)** \| **%** \| **Kzt (CI 95%)** \| **%** \| **Kzt (CI 95%)** \| **%** \| **Kzt (CI 95%)** \| **%** \| **Kzt (CI 95%)** \| **%** \| **Kzt (CI 95%)** \| **%** \| **Kzt (CI 95%)** \| \| **Women** \| \|  \|  \| 24.0 \|  \| 43.2 \|  \| 48.0 \|  \| 30.1 \|  \| 43.7 \|  \| 24.5 \|  \| 15.2 \|  \| \| **Age groups** \| \|  \|  \|  \|  \|  \|  \|  \|  \|  \|  \|  \|  \|  \|  \|  \|  \| \|  \| < 25 \|  \|  \| 10.7 \| **1** \| 35.4 \| **1** \| 35.5 \| **1** \| 13.8 \| **1** \| 29.3 \| **1** \| 6.5 \| **1** \| 4.8 \| **1** \| \|  \| 25-44 \|  \|  \| 20.6 \| 1.9 (0.9 - 4.3) \| 38.6 \| 1.1 (0.9 - 1.4) \| 45.1 \| 1.3 (0.9 - 1.7) \| 27.9 \| 2 (1.1 - 3.6) \| 39.0 \| 1,3 (1,1 - 1,6) \| 20.5 \| 3,2 (1,2 - 8,4) \| 14.4 \| 3 (1,2 - 7,2) \| \|  \| 45-64 \|  \|  \| 38.0 \| 3.5 (1.6 - 8) \| 56.2 \| 1.6 (1.2 - 2) \| 60.3 \| 1.7 (1.2 - 2.3) \| 38.6 \| 2.8 (1.6 - 5) \| 55.1 \| 1,9 (1,5 - 2,3) \| 30.3 \| 4,7 (1,8 - 12,3) \| 19.5 \| 4 (1,7 - 9,7) \| \|  \| >65 \|  \|  \| 85.7 \| 8 (3.5 - 18.1) \| 79.2 \| 2.2 (1.7 - 3) \| 75.0 \| 2.1 (1.4 - 3.1) \| 71.4 \| 5.2 (2.7 - 9.9) \| 87.8 \| 3 (2,5 - 3,6) \| 43.3 \| 6,7 (2,4 - 18,9) \| 21.7 \| 4,5 (1,4 - 14,9) \| \| **Educational level** \| \|  \|  \|  \|  \|  \|  \|  \|  \|  \|  \|  \|  \|  \|  \|  \|  \| \|  \| High \|  \|  \| 9.9 \| **1** \| 20.3 \| **1** \| 33.0 \| **1** \| 25.6 \| **1** \| 32.1 \| **1** \| 21.9 \| **1** \| 9.3 \| **1** \| \|  \| Middle \|  \|  \| 19.6 \| 2 (0.9 - 4.3) \| 30.9 \| 1.5 (0.9 - 2.6) \| 44.0 \| 1.3 (1 - 1.8) \| 25.0 \| 1 (0.7 - 1.4) \| 41.6 \| 1,3 (1,1 - 1,5) \| 34.9 \| 1,6 (1,2 - 2,1) \| 15.9 \| 1,7 (1,1 - 2,6) \| \|  \| Low \|  \|  \| 42.2 \| 4.3 (2 - 9) \| 54.9 \| 2.7 (1.6 - 4.5) \| 57.3 \| 1.7 (1.3 - 2.4) \| 54.4 \| 2.1 (1.5 - 3) \| 59.4 \| 1,8 (1,6 - 2,2) \| 62.5 \| 2,9 (1,6 - 5) \| 26.1 \| 2,8 (1,8 - 4,3) \| \|  \| >Low \|  \|  \| 80.0 \| 8.1 (3.5 - 18.6) \| 58.8 \| 2.9 (1.6 - 5.2) \| 65.1 \| 2 (1.4 - 2.8) \| 66.7 \| 2.6 (1.1 - 6) \| 77.9 \| 2,4 (2,1 - 2,9) \|  \|  \| 26.9 \| 2,9 (0,6 - 13) \| \| **Occupational categories** \| \| \|  \|  \|  \|  \|  \|  \|  \|  \|  \|  \|  \|  \|  \|  \|  \| \|  \| Non-manual skilled \|  \|  \| 6.7 \| **1** \| 37.2 \| **1** \| 37.9 \| **1** \| 24.7 \| **1** \| 28.1 \| **1** \| - \| **-** \| 9.7 \| **1** \| \|  \| Non-nanual non-skilled \| \|  \| 16.3 \| 2.4 (0.7 - 8.8) \| 42.9 \| 1.2 (0.8 - 1.7) \| 44.9 \| 1.2 (0.8 - 1.7) \| 23.7 \| 1 (0.6 - 1.5) \| 41.5 \| 1,5 (1,2 - 1,8) \| - \| 0 \| 12.8 \| 1,3 (0,8 - 2,1) \| \|  \| Manual skilled \|  \|  \| 19.0 \| 2.9 (0.9 - 9.3) \| 43.9 \| 1.2 (0.8 - 1.8) \| 59.9 \| 1.6 (1.1 - 2.2) \| 43.4 \| 1.8 (1.1 - 2.7) \| 52.3 \| 1,9 (1,4 - 2,4) \| - \| 0 \| 15.0 \| 1,6 (0,6 - 4,3) \| \|  \| Manual non-skilled \|  \|  \| 33.1 \| 5.0 (1.6 - 15.2) \| 44.4 \| 1.2 (0.8 - 1.8) \| 40.0 \| 1.1 (0.7 - 1.6) \| 35.5 \| 1.4 (0.9 - 2.3) \| 57.8 \| 2,1 (1,7 - 2,6) \| - \| 0 \| 24.8 \| 2,6 (1,6 - 4) \| \| **Men** \| \|  \|  \| 22.9 \|  \| 43.8 \|  \| 43.4 \|  \| 22.9 \|  \| 34.5 \|  \| 18.1 \|  \| 9.2 \|  \| \| **Age groups** \| \|  \|  \|  \|  \|  \|  \|  \|  \|  \|  \|  \|  \|  \|  \|  \|  \| \|  \| < 25 \|  \|  \| 18.5 \| **1** \| 18.8 \| **1** \| 27.6 \| **1** \| 11.8 \| **1** \| 20.6 \| **1** \| 7.7 \| **1** \| 7.4 \| **1** \| \|  \| 25-44 \|  \|  \| 17.0 \| 0.9 (0.6 - 1.4) \| 38.8 \| 2.1 (1.5 - 2.8) \| 41.7 \| 1.5 (1.2 - 1.9) \| 16.4 \| 1.4 (0.9 - 2.2) \| 29.7 \| 1,4 (1,1 - 1,8) \| 12.6 \| 1,6 (0,7 - 4) \| 6.4 \| 0,9 (0,4 - 1,8) \| \|  \| 45-64 \|  \|  \| 36.2 \| 2 (1.3 - 3) \| 61.4 \| 3.3 (2.4 - 4.4) \| 59.0 \| 2.1 (1.7 - 2.8) \| 32.0 \| 2.7 (1.7 - 4.2) \| 46.4 \| 2,3 (1,8 - 2,8) \| 24.0 \| 3,1 (1,3 - 7,4) \| 13.6 \| 1,9 (0,9 - 3,7) \| \|  \| >65 \|  \|  \| 46.4 \| 2.5 (1.5 - 4.3) \| 75.0 \| 4 (2.9 - 5.4) \| 70.8 \| 2.6 (1.8 - 3.6) \| 57.1 \| 4.9 (3 - 7.8) \| 55.5 \| 2,7 (2,1 - 3,5) \| 31.7 \| 4,1 (1,6 - 10,7) \| 10.6 \| 1,4 (0,4 - 4,6) \| \| **Educational level** \| \|  \|  \|  \|  \|  \|  \|  \|  \|  \|  \|  \|  \|  \|  \|  \|  \| \|  \| High \|  \|  \| 44.4 \| **1** \| 26.8 \| **1** \| 31.7 \| **1** \| 16.3 \| **1** \| 26.0 \| **1** \| 12.5 \| **1** \| 6.9 \| **1** \| \|  \| Middle \|  \|  \| 38.5 \| 1.9 (1.1 - 3.2) \| 24.2 \| 0.9 (0.5 - 1.6) \| 37.1 \| 1.2 (0.9 - 1.5) \| 16.1 \| 1 (0.6 - 1.5) \| 34.2 \| 1,3 (1,1 - 1,6) \| 27.9 \| 2,2 (1,6 - 3) \| 8.5 \| 1,2 (0,7 - 2,1) \| \|  \| Low \|  \|  \| 20.7 \| 3.5 (2.1 - 5.9) \| 52.3 \| 2 (1.2 - 3.3) \| 49.9 \| 1.6 (1.2 - 2) \| 38.1 \| 2.3 (1.6 - 3.5) \| 47.2 \| 1,8 (1,5 - 2,2) \| 36.7 \| 2,9 (1,7 - 4,9) \| 13.3 \| 1,9 (1,1 - 3,3) \| \|  \| >Low \|  \|  \| 10.9 \| 4.1 (2 - 8.2) \| 62.7 \| 2.3 (1.4 - 4) \| 56.9 \| 1.8 (1.3 - 2.4) \| 40.7 \| 2.5 (1.5 - 4.1) \| 64.0 \| 2,5 (2 - 3,1) \|  \|  \| 34.0 \| 4,9 (1,7 - 14,1) \| \| **Occupational categories** \| \| \|  \|  \|  \|  \|  \|  \|  \|  \|  \|  \|  \|  \|  \|  \|  \| \|  \| Non-manual skilled \|  \|  \| 18.2 \| 1 \| 23.6 \| **1** \| 30.0 \| **1** \| 16.7 \| **1** \| 24.7 \| **1** \| - \| **-** \| 5.8 \| **1** \| \|  \| Non-nanual non-skilled \| \|  \| 16.2 \| 0.9 (0.4 - 1.8) \| 33.7 \| 1.4 (0.8 - 2.5) \| 36.4 \| 1.2 (0.8 - 1.9) \| 25.2 \| 1.5 (0.8 - 2.7) \| 29.6 \| 1,2 (1 - 1,5) \| - \| 0 \| 8.4 \| 1,4 (0,8 - 2,8) \| \|  \| Manual skilled \|  \|  \| 28.4 \| 1.6 (0.9 - 2.6) \| 47.1 \| 2 (1.2 - 3.2) \| 45.3 \| 1.5 (1 - 2.2) \| 24.1 \| 1.4 (0.8 - 2.5) \| 40.9 \| 1,7 (1,3 - 2) \| - \| 0 \| 7.5 \| 1,3 (0,6 - 2,7) \| \|  \| Manual non-skilled \|  \|  \| 22.5 \| 1.2 (0.8 - 2) \| 45.6 \| 1.9 (1.2 - 3.2) \| 46.1 \| 1.5 (1 - 2.3) \| 20.2 \| 1.2 (0.7 - 2.2) \| 42.8 \| 1,7 (1,4 - 2,2) \| - \| 0 \| 16.1 \| 2,8 (1,5 - 5,1) \| \| % prevalence of poor self-perceived health \| \| \| \|  \|  \|  \|  \|  \|  \|  \|  \|  \|  \|  \|  \|  \|  \| \| Kzt Kuznets relative index \| \| \|  \|  \|  \|  \|  \|  \|  \|  \|  \|  \|  \|  \|  \|  \|  \| \| CI 95% Confindence Interval \| \| \|  \|  \|  \|  \|  \|  \|  \|  \|  \|  \|  \|  \|  \|  \|  \| |  |  |  |  |  |  |  |  |  |
| --- | --- | --- | --- | --- | --- | --- | --- | --- | --- | --- | --- | --- | --- | --- | --- | --- | --- | --- | --- | --- | --- | --- | --- | --- | --- | --- | --- | --- | --- | --- | --- | --- | --- | --- | --- | --- | --- | --- | --- | --- | --- | --- | --- | --- | --- | --- | --- | --- | --- | --- | --- | --- | --- | --- | --- | --- | --- | --- | --- | --- | --- | --- | --- | --- | --- | --- | --- | --- | --- | --- | --- | --- | --- | --- | --- | --- | --- | --- | --- | --- | --- | --- | --- | --- | --- | --- | --- | --- | --- | --- | --- | --- | --- | --- | --- | --- | --- | --- | --- | --- | --- | --- | --- | --- | --- | --- | --- | --- | --- | --- | --- | --- | --- | --- | --- | --- | --- | --- | --- | --- | --- | --- | --- | --- | --- | --- | --- | --- | --- | --- | --- | --- | --- | --- | --- | --- | --- | --- | --- | --- | --- | --- | --- | --- | --- | --- | --- | --- | --- | --- | --- | --- | --- | --- | --- | --- | --- | --- | --- | --- | --- | --- | --- | --- | --- | --- | --- | --- | --- | --- | --- | --- | --- | --- | --- | --- | --- | --- | --- | --- | --- | --- | --- | --- | --- | --- | --- | --- | --- | --- | --- | --- | --- | --- | --- | --- | --- | --- | --- | --- | --- | --- | --- | --- | --- | --- | --- | --- | --- | --- | --- | --- | --- | --- | --- | --- | --- | --- | --- | --- | --- | --- | --- | --- | --- | --- | --- | --- | --- | --- | --- | --- | --- | --- | --- | --- | --- | --- | --- | --- | --- | --- | --- | --- | --- | --- | --- | --- | --- | --- | --- | --- | --- | --- | --- | --- | --- | --- | --- | --- | --- | --- | --- | --- | --- | --- | --- | --- | --- | --- | --- | --- | --- | --- | --- | --- | --- | --- | --- | --- | --- | --- | --- | --- | --- | --- | --- | --- | --- | --- | --- | --- | --- | --- | --- | --- | --- | --- | --- | --- | --- | --- | --- | --- | --- | --- | --- | --- | --- | --- | --- | --- | --- | --- | --- | --- | --- | --- | --- | --- | --- | --- | --- | --- | --- | --- | --- | --- | --- | --- | --- | --- | --- | --- | --- | --- | --- | --- | --- | --- | --- | --- | --- | --- | --- | --- | --- | --- | --- | --- | --- | --- | --- | --- | --- | --- | --- | --- | --- | --- | --- | --- | --- | --- | --- | --- | --- | --- | --- | --- | --- | --- | --- | --- | --- | --- | --- | --- | --- | --- | --- | --- | --- | --- | --- | --- | --- | --- | --- | --- | --- | --- | --- | --- | --- | --- | --- | --- | --- | --- | --- | --- | --- | --- | --- | --- | --- | --- | --- | --- | --- | --- | --- | --- | --- | --- | --- | --- | --- | --- | --- | --- | --- | --- | --- | --- | --- | --- | --- | --- | --- | --- | --- | --- | --- | --- | --- | --- | --- | --- | --- | --- | --- | --- | --- | --- | --- | --- | --- | --- | --- | --- | --- | --- | --- | --- | --- | --- | --- | --- | --- | --- | --- | --- | --- | --- | --- | --- | --- | --- | --- | --- | --- | --- | --- | --- | --- | --- | --- | --- | --- | --- | --- | --- | --- | --- | --- | --- | --- | --- | --- | --- | --- | --- | --- | --- | --- | --- | --- | --- | --- | --- | --- | --- | --- | --- | --- | --- | --- | --- | --- | --- | --- | --- | --- | --- | --- | --- | --- | --- | --- | --- | --- | --- | --- | --- | --- | --- | --- | --- | --- | --- | --- | --- | --- | --- | --- | --- | --- | --- | --- | --- | --- | --- | --- | --- | --- | --- | --- | --- | --- | --- | --- | --- | --- | --- | --- | --- | --- | --- | --- | --- | --- | --- | --- | --- | --- | --- | --- | --- | --- | --- | --- | --- | --- | --- | --- | --- | --- | --- | --- | --- | --- | --- | --- | --- | --- | --- | --- | --- | --- | --- | --- | --- | --- | --- | --- | --- | --- | --- | --- | --- | --- | --- | --- | --- | --- | --- | --- | --- | --- | --- | --- | --- | --- | --- | --- | --- | --- | --- | --- | --- | --- | --- | --- | --- | --- | --- | --- | --- | --- | --- | --- | --- | --- | --- | --- | --- | --- | --- | --- | --- | --- | --- | --- | --- | --- | --- | --- | --- | --- | --- | --- | --- | --- | --- | --- | --- | --- | --- | --- | --- | --- | --- | --- | --- | --- | --- | --- | --- | --- | --- | --- | --- | --- | --- | --- | --- | --- | --- | --- | --- | --- | --- | --- | --- | --- | --- | --- | --- | --- | --- | --- | --- | --- | --- | --- | --- | --- | --- | --- | --- | --- | --- | --- | --- | --- | --- | --- | --- | --- | --- | --- | --- | --- | --- | --- | --- | --- | --- | --- | --- | --- | --- | --- | --- | --- | --- | --- | --- | --- | --- | --- | --- | --- | --- | --- | --- | --- | --- | --- | --- | --- | --- | --- | --- | --- | --- | --- | --- | --- | --- | --- | --- | --- | --- | --- | --- | --- | --- | --- | --- | --- | --- | --- | --- | --- | --- | --- | --- | --- | --- | --- | --- | --- | --- | --- | --- | --- | --- | --- | --- | --- | --- | --- | --- | --- | --- | --- | --- | --- | --- | --- | --- | --- | --- | --- | --- | --- | --- | --- | --- | --- | --- | --- | --- | --- | --- | --- | --- | --- | --- | --- | --- | --- | --- | --- | --- | --- | --- | --- | --- | --- | --- | --- | --- | --- | --- | --- | --- | --- | --- | --- | --- | --- | --- | --- | --- | --- | --- | --- | --- | --- | --- | --- | --- | --- | --- | --- | --- | --- | --- | --- | --- | --- | --- | --- | --- | --- | --- | --- | --- | --- | --- | --- | --- | --- | --- | --- | --- | --- | --- | --- | --- | --- | --- | --- | --- | --- | --- | --- | --- | --- | --- | --- | --- | --- | --- | --- | --- | --- | --- | --- | --- | --- | --- | --- | --- | --- | --- | --- | --- | --- | --- | --- | --- | --- | --- | --- | --- | --- | --- | --- | --- | --- | --- | --- | --- | --- | --- | --- | --- | --- | --- | --- | --- | --- | --- | --- | --- | --- | --- | --- | --- | --- | --- | --- | --- | --- | --- | --- | --- | --- | --- | --- | --- | --- | --- | --- | --- | --- | --- | --- | --- | --- | --- | --- | --- | --- | --- | --- | --- | --- | --- | --- | --- | --- | --- | --- | --- | --- | --- | --- | --- | --- | --- | --- | --- | --- | --- | --- | --- | --- | --- | --- | --- | --- | --- | --- | --- | --- | --- | --- | --- | --- | --- | --- | --- | --- | --- | --- | --- | --- | --- | --- | --- | --- | --- | --- | --- | --- | --- | --- | --- | --- | --- | --- | --- | --- | --- | --- | --- | --- | --- | --- | --- | --- | --- | --- | --- | --- | --- | --- | --- | --- | --- | --- | --- | --- | --- | --- | --- | --- | --- | --- | --- | --- | --- | --- | --- | --- | --- | --- | --- | --- | --- | --- | --- | --- | --- | --- | --- | --- | --- | --- | --- | --- | --- | --- | --- | --- | --- | --- | --- | --- | --- | --- | --- | --- | --- | --- | --- | --- | --- | --- | --- | --- | --- | --- | --- | --- | --- | --- | --- | --- | --- | --- | --- | --- | --- | --- | --- | --- | --- | --- | --- | --- | --- | --- | --- | --- | --- | --- | --- | --- | --- | --- | --- | --- | --- | --- | --- | --- | --- | --- | --- | --- | --- | --- | --- | --- | --- | --- | --- | --- | --- | --- | --- | --- | --- | --- | --- | --- | --- | --- | --- | --- | --- | --- | --- | --- | --- | --- | --- | --- | --- | --- | --- | --- | --- | --- | --- | --- | --- | --- | --- | --- | --- | --- | --- | --- | --- | --- | --- | --- | --- | --- | --- | --- | --- | --- | --- | --- | --- | --- | --- | --- | --- | --- | --- | --- | --- | --- | --- | --- | --- | --- | --- | --- | --- | --- | --- | --- | --- | --- | --- | --- | --- | --- | --- | --- | --- | --- | --- | --- | --- | --- | --- | --- | --- | --- | --- | --- | --- | --- | --- | --- | --- | --- | --- | --- | --- | --- | --- | --- | --- | --- | --- | --- | --- | --- | --- | --- | --- | --- | --- | --- | --- | --- | --- | --- | --- | --- | --- | --- | --- | --- | --- | --- | --- | --- | --- | --- | --- | --- | --- | --- | --- | --- | --- | --- | --- | --- | --- | --- | --- | --- | --- | --- | --- | --- | --- | --- | --- | --- | --- | --- | --- | --- | --- | --- | --- | --- | --- | --- | --- | --- | --- | --- | --- | --- | --- | --- | --- | --- | --- | --- | --- | --- | --- | --- | --- | --- | --- | --- | --- | --- | --- | --- | --- | --- | --- | --- | --- | --- | --- | --- | --- | --- | --- | --- | --- | --- | --- | --- | --- | --- | --- | --- | --- | --- | --- | --- | --- | --- | --- | --- | --- | --- | --- | --- | --- | --- | --- | --- | --- | --- | --- | --- | --- | --- | --- | --- | --- | --- | --- | --- | --- | --- | --- | --- | --- | --- | --- | --- | --- | --- |
